# Studies in *Lepiota* (*Agaricales*, *Verrucosporaceae*): supporting the merger of *Chamaemyces* into *Lepiota* and proposing two new species

**DOI:** 10.3897/mycokeys.133.186351

**Published:** 2026-05-28

**Authors:** Xing Li, Bin Chen, Guohua Qu, Mengya An, Junfeng Liang

**Affiliations:** 1 Research Institute of Tropical Forestry, Chinese Academy of Forestry, Guangzhou 510520, China Research Institute of Tropical Forestry, Chinese Academy of Forestry Guangzhou China; 2 College of Forestry, Nanjing Forestry University, Nanjing 210037, China College of Forestry, Nanjing Forestry University Nanjing China; 3 Henan Institute of Science and Technology, College of Life Science, Xinxiang 453003, China Henan Institute of Science and Technology, College of Life Science Xinxiang China

**Keywords:** China, new species, phylogeny, synonymy, taxonomy

## Abstract

The taxonomic status of *Chamaemyces* as a genus separate from *Lepiota* has recently been questioned, as its type species has been transferred to *Lepiota*, implicitly rendering the merger of *Chamaemyces* as a synonym without a resolved sectional assignment or morphological validation. Multilocus (ITS, LSU, *rpb*2, mtSSU) phylogenetic analyses robustly place this transferred species within *L.* sect. *Cristatae*, and detailed morphological re-evaluation further supports the merger. The recently proposed seven-section classification of *Lepiota* is largely supported by the phylogenetic framework, which also clarifies the phylogenetic position of *L. angusticystidiata*. Furthermore, two novel species are described and illustrated: *L. pallidovelata* (sect. *Lepiota*), which is characterized by whitish squamules on the pileus with veil remnants, and *L. stillispora* (sect. *Helveolae*), distinguished by its droplet-shaped basidiospores. Both the sectional assignment and the proposal of novel species were further independently confirmed by a comprehensive ITS phylogeny of the genus. This study provides conclusive phylogenetic and morphological evidence for the prior merger and expands the known diversity of *Lepiota* in East Asia with two new taxa.

## Introduction

The family *Verrucosporaceae* represents a diverse and taxonomically complex group within the order *Agaricales*, currently encompassing eight recognized genera (Yang et al. 2024a; Salichanh et al. 2024; Li et al. 2025a), viz., *Lepiota* (Pers.) Gray (1821), *Pulverolepiota*Bon (1993), *Melanophyllum* Velen. (1921), *Cystolepiota* Singer (Singer and Digilio 1952), *Echinoderma*Bon (1991), *Smithiomyces*Singer (1944), *Chamaemyces* Battarra ex Earle (1909), and *Verrucospora* E. Horak (1967). Among these, the genus *Lepiota* is particularly significant due to its species richness, morphological diversity, wide distribution from tropical to temperate regions (Vellinga 2004a; Sysouphanthong et al. 2011), and historical role as a taxonomic repository for numerous lepiotaceous fungi. The delimitation of *Lepiota* and its satellite genera has undergone considerable revision (Lange 1915, 1935; Kühner 1936; Singer 1943, 1951, 1975, 1986; Singer and Digilio 1952; Locquin 1945; Horak 1967; Knudsen 1978, 1980; Wasser 1978; Bon 1981, 1991, 1993, 1996; Candusso and Lanzoni 1990; Vellinga 2001; Yang et al. 2019), especially with the introduction of molecular phylogenies (Vellinga 2003, 2004b; Hou and Ge 2020; Ge et al. 2021; Qu et al. 2023). Recent large-scale phylogenetic studies based on multilocus datasets (ITS, LSU, *rpb*2, mtSSU) have been instrumental in refining the infrageneric classification of *Lepiota*, leading to the recognition of seven sections, viz., sect. *Stenosporae* J.E. Lange ex Reschke & Sarawi, sect. *Helveolae* (Bon & Boiffard) Bon (1993), sect. *Cristatae* Kühner ex Wasser (1978), sect. *Fuscovinaceae* Bon & Candusso (Candusso and Lanzoni 1990), sect. *Lepiota*, sect. *Lilaceae* Bon, (1981), and sect. *Eriophorae* (Bon) Reschke & Sarawi (Sarawi et al. 2025a). Such a phylogenetic framework has resolved long-standing polyphyletic sections, e.g., the former sect. *Ovisporae* (Lange, 1935, nom. inval.) and has accommodated sequestrate forms and species with divergent morphologies within a monophyletic *Lepiota*. However, the taxonomic status of several smaller genera within *Verrucosporaceae*, particularly *Chamaemyces*, remains ambiguous and requires formal reassessment. This genus can traditionally be distinguished from *Lepiota* by its viscid pileus, small droplets on the stipe, metachromatic spore walls in cresyl blue, the presence of pleurocystidia, and a distinctively monovelangiocarpic and stipitocarpic developmental mode (Reijnders 1975; Vellinga 2001, 2003; Didukh et al. 2004; Yang et al. 2019). Significantly, several recent studies (Yang et al. 2024a, 2024b; Li et al. 2025a) based on multilocus datasets (ITS, LSU, *rpb*2, *tef*1-α) have placed its type species, *C. fracidus* (Fr.) Donk, within *Lepiota*, and Li et al. (2025a) formally proposed the new combination *L. fracida* (Fr.) R.L. Zhao & J.X. Li. This taxonomic treatment implicitly results in the merger of *Chamaemyces* into *Lepiota*, making the former a synonym of the latter. However, this merger not only lacked explicit morphological validation but also left the precise sectional placement of *L. fracida* unresolved due to limited sampling and the absence of key loci for some species. A comprehensive phylogenetic framework and morphological re-evaluation of *L. fracida* are thus required to definitively assign this species to one of the recognized sections within *Lepiota*. Concurrently, ongoing regional studies in China continue to reveal novel *Lepiota* diversity (e.g., Zhang et al. 2019; Hou and Ge 2020; Mao et al. 2023; Liang et al. 2023; Li et al. 2025b). These discoveries are essential for completing regional inventories and for testing and refining the phylogenetic framework of the genus.

To address these gaps, this study employs an expanded multilocus phylogenetic analysis based on the same datasets (ITS, LSU, *rpb*2, and mtSSU) as those of Sarawi et al. (2025a) for *Lepiota*, accompanied by a comparative phylogeny based on the same datasets (ITS, LSU, *rpb*2, and *tef*1-α) as those of Li et al. (2025a) and a comprehensive ITS phylogeny for further validation. This study set three specific aims: (1) to evaluate the current seven-section classification and clarify the sectional placement of *L. fracida*; (2) to discuss in depth the placement of *L. fracida* via phylogenetic results and detailed morphological re-evaluation to further support the prior merger of *Chamaemyces* proposed by Li et al. (2025a); and (3) to describe and illustrate two new species from China belonging to *L.* sect. *Lepiota* and *Helveolae*, respectively.

## Materials and methods

### Morphological studies

Specimens in this study were collected from five provinces or municipalities (Jilin, Yunnan, Beijing, Guangdong, and Zhejiang). Fresh basidiomata were stored in sealed bags containing silica gel for subsequent DNA extraction. Dry voucher specimens were deposited in the following herbaria, viz., the Herbarium of Research Institute of Tropical Forestry, Chinese Academy of Forestry (**RITF**), Guangzhou, Guangdong, the Herbarium of Jilin Agricultural University (**HMJAU**), Changchun, Jilin, the Herbarium of Cryptogams, and Kunming Institute of Botany, Chinese Academy of Sciences (**HKAS**), Kunming, Yunnan. Macromorphological features were documented from field observations, photographs, and measurements of fresh basidiomata, as well as dry specimens. The color designations of basidiomata were described with reference to Kornerup and Wanscher (1981). Descriptive terminology follows Vellinga (1988), while herbarium acronyms follow Thiers (2026).

Microstructures and dimensions were examined and quantified using a ZEISS Imager M2 microscope at 1000× magnification, following the methods of Yang et al. (2019). Specimen sections (20–50 μm) obtained by manual slicing were fixed in 5% KOH and stained with Congo red or Melzer’s reagent for microscopic observation. Moreover, basidiospores were immersed in cresyl blue to assess metachromatic reactions. For each morphological character per collection, a minimum of 20 elements were randomly measured. The notation [*n*/*m*/*p*] denotes measurements from “*n*” basidiospores derived from “*m*” basidiomata across “*p*” specimens. The size of basidiospores is presented as the symbol “(*a*) *b*–*c* (*d*),” where values “*b*” to “*c*” encompass 90% of the data range, while “*a*” and “*d*” represent the extreme values. The length-width ratio of a single basidiospore is expressed as “*Q*,” and “*Q*av” refers to the average value of “*Q*” of all sampled specimens.

### DNA extraction, amplification, and sequencing

Total genomic DNA was extracted from silica-dried samples using the Fungi DNA Kit (Aidlab Biotechnologies Co., Ltd., Beijing, China). The following four DNA regions were amplified by polymerase chain reaction (PCR) using the specified primer pairs: ITS1F (Gardes and Bruns 1993) and ITS4 (White et al. 1990) for the nuclear ribosomal internal transcribed spacer (nrITS), LR0R and LR7 (Vilgalys and Hester 1990) for the 28S nuclear ribosomal large subunit region (nrLSU), bRPB2-6F and bRPB2-7.1R (Matheny 2005) for the second-largest subunit of RNA polymerase II (*rpb*2), and MS3/MR4 (Liang et al. 2009) for the mitochondrial small subunit region (mtSSU). The PCR products were then purified and subjected to bidirectional sequencing by Novogene (Beijing, China).

### Phylogenetic analysis

Three datasets were constructed for phylogenetic analyses. First, a combined four-locus dataset (ITS, LSU, *rpb*2, mtSSU) identical to that used by Sarawi et al. (2025a) was constructed (hereafter referred to as the mtSSU-based phylogeny). Taxon sampling for this matrix prioritized sequences from type specimens and was limited to taxa with available data for at least three of the four loci, with final representation of 80 taxa for ITS, 80 for LSU, 73 for *rpb*2, and 70 for mtSSU, thereby resulting in 80 representative taxa. This sampling comprised species from all seven sections of *Lepiota* as circumscribed by Sarawi et al. (2025a), with three specimens of *Echinoderma asperum* (Pers.) Bon as the outgroup. Second, an ITS-only dataset (hereafter referred to as the ITS phylogeny) was constructed to place the new findings within the broadest taxonomic framework. It aimed to include all publicly available ITS sequences from the described species of *Lepiota* for the present analysis (totaling 176 taxa), thereby maximizing representation across the genus. Additionally, another combined four-locus dataset (ITS, LSU, *rpb*2, *tef*1-α) was constructed (hereafter referred to as the *tef*1-α-based phylogeny) for comparative phylogenetic analysis with that of Li et al. (2025a), who used the same dataset to transfer *C. fracidus* to *Lepiota*, with taxon sampling following the same criterion as that of the mtSSU-based phylogeny (83 taxa for ITS, 81 for LSU, 82 for *rpb*2, and 29 for *tef*1-α). All sequence data have been deposited in GenBank, with corresponding accession numbers, voucher specimen details, and taxonomic information provided in Suppl. material 1. The sequences obtained in this study are indicated in bold, with the holotype and paratype represented by HT and PT, respectively.

The four DNA fragments (ITS, LSU, *rpb*2, mtSSU, or *tef*1-α) were aligned using MAFFT v7.490 (Katoh and Standley 2013) and merged into the corresponding combined data matrix. Phylogenetic analyses were conducted using both maximum likelihood (ML) and Bayesian inference (BI) approaches, implemented in PhyloSuite v2 (Zhang et al. 2025). The optimal partitioning scheme and substitution models for each DNA region were determined using ModelFinder v2.2.0 (Kalyaanamoorthy et al. 2017) under the Bayesian information criterion (BIC), with edge-linked partitioning applied for both ML and BI analyses. ML analysis was performed using IQ-TREE v2.2.0 (Minh et al. 2020), with nodal support evaluated through 5000 ultrafast bootstrap replicates (Minh et al. 2013) and 1000 Shimodaira-Hasegawa approximate likelihood ratio tests (SH-aLRT) (Guindon et al. 2010). The BI analysis was performed using MrBayes v3.2.7 (Ronquist et al. 2012). Two simultaneous Markov chain Monte Carlo (MCMC) runs were conducted with 40 million generations each, sampling trees every 1000 generations. To ensure convergence, the initial 25% of samples were discarded as burn-in before summarizing the posterior distribution of trees. The phylogenetic trees were visualized and annotated using the interactive Tree of Life (iTOL) online tool (https://itol.embl.de/).

## Result

### Phylogenetic analysis

**Figure 1.**
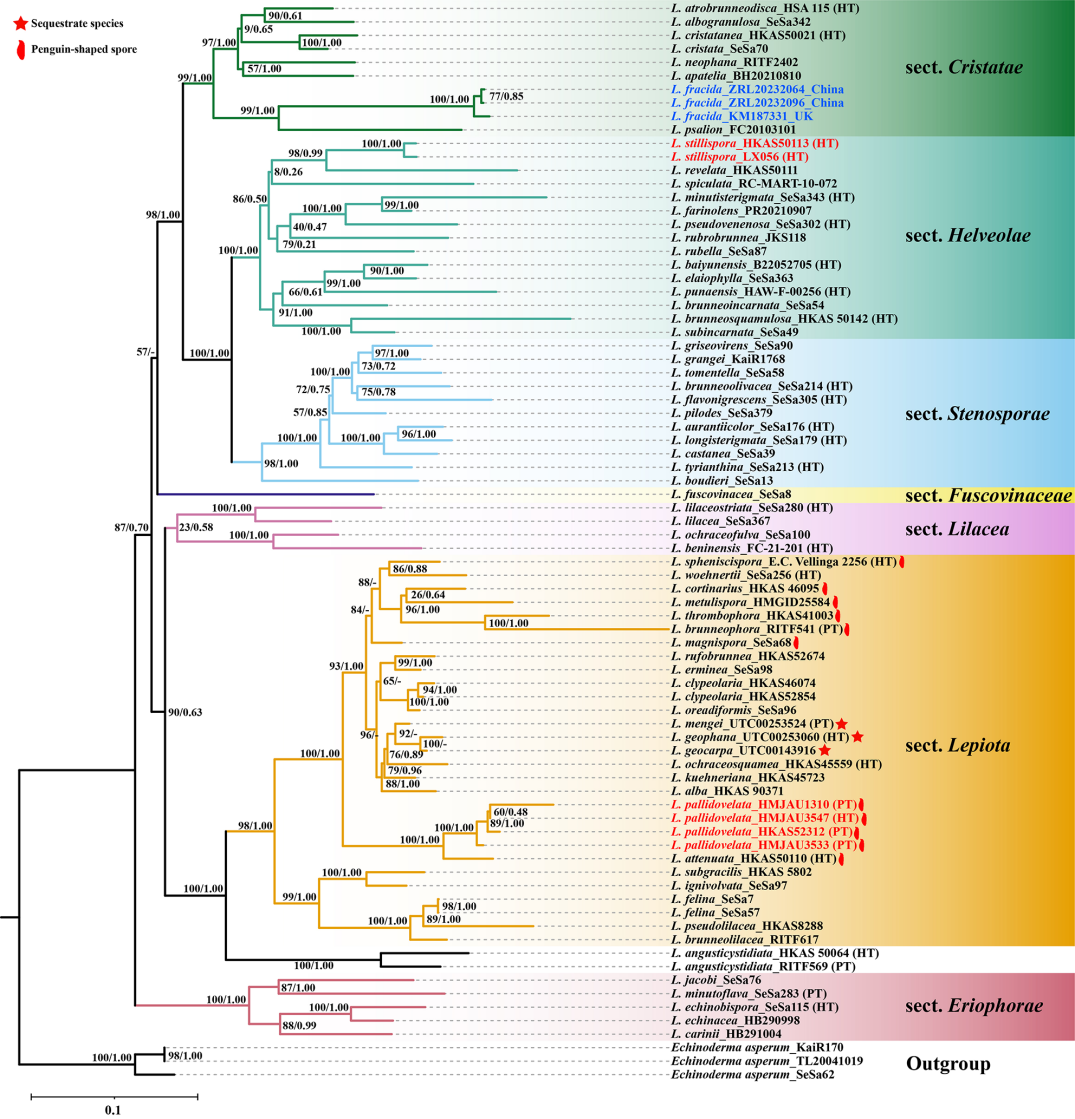
Phylogenetic placement of *Lepiota fracida* (≡ *Chamaemyces fracidus*) and two new species within *Lepiota*. The maximum likelihood tree is inferred from a combined dataset of ITS, LSU, *rpb*2, and mtSSU sequences. Branches are labeled with bootstrap support values (left) and Bayesian posterior probabilities (right). Newly proposed taxa are in bold red, and the newly transferred *L. fracida* is in bold blue. Holotype and paratype are indicated by HT and PT, respectively.

The concatenated dataset of the mtSSU-based phylogeny yielded an alignment (Suppl. material 2) of 4896 bp, with partitioned loci as follows: ITS (1–1226), LSU (1227–2679), *rpb*2 (2680–3695), and mtSSU (3696–4896). Sequence analysis revealed 1,723 variable sites (35.20%), consisting of 555 singleton variable sites (11.34%) and 1168 parsimony-informative sites (23.86%). Phylogenetic reconstructions using both ML (ITS: TPM2u+F+I+I+R3, LSU: SYM+I+I+R3, *rpb*2: TIM2e+I+I+R3, mtSSU: GTR+F+I+G4) and BI (ITS: GTR+F+I+G4, LSU: SYM+I+G4, *rpb*2: SYM+G4, mtSSU: GTR+F+I+G4) approaches produced similar topologies. The ML tree (Fig. 1; Suppl. materials 3, 4) is shown as the representative phylogeny, with branches annotated with ML bootstrap support (BS) values and BI posterior probabilities (PP).

The mtSSU-based phylogeny (Fig. 1) robustly recovered the seven major clades, corresponding to the sections of *Lepiota* recently recognized by Sarawi et al. (2025a), with an overall topology largely congruent with their framework. As in their study, *L.* sect. *Eriophorae* (BS = 100% and PP = 1.00) occupies a basal position within the genus. A prominent, well-supported clade (BS = 98% and PP = 1.00) comprises sect. *Cristatae* (BS = 99% and PP = 1.00), *Helveolae* (BS = 100% and PP = 1.00), and *Stenosporae* (BS = 98% and PP = 1.00). Within this clade, sect. *Helveolae* and *Stenosporae* (BS = 100% and PP = 1.00) are sister groups with sect. *Cristatae* positioned alongside them. However, this analysis revealed a notable difference in the phylogenetic position of *L. angusticystidiata* Jun F. Liang & Zhu L. Yang. In the topology, sect. *Lepiota* (BS = 98% and PP = 1.00) and two samples of *L. angusticystidiata* (BS = 100% and PP = 1.00) form a fully supported clade (BS = 100% and PP = 1.00) that is sister to sect. *Lilaceae* (BS = 90% and PP = 0.63), whereas Sarawi et al. (2025a) recovered sect. *Lilaceae* and sect. *Lepiota* as a joint clade without the inclusion of *L. angusticystidiata*.

The *tef*1-α-based phylogeny (Suppl. material 5), constructed for comparative analysis, aligns with the mtSSU-based phylogeny in several key topological aspects. Section *Eriophorae* maintains the basal position with full statistical support (BS = 100% and PP = 1.00). Sections *Cristatae* (BS = 81% and PP = 0.95), *Helveolae* (BS = 98% and PP = 0.96), and *Stenosporae* (BS = 99% and PP = 1.00) form a well-supported clade (BS = 90% and PP = 0.90), with the latter two sections resolved as sister groups (BS = 100% and PP = 1.00) within this clade. *Lepiota angusticystidiata* and sect. *Lepiota* also form a fully supported clade (BS = 98% and PP = 1.00), with sect. *Lepiota* itself receiving strong support (BS = 99% and PP = 1.00). However, sects. *Lilaceae* and *Fuscovinaceae* exhibit unstable positions due to insufficient statistical support.

Significantly, in both the mtSSU-based and *tef*1-α-based phylogenies, *L. fracida* is robustly placed within sect. *Cristatae*. Sequences from two Asian (ZRL20232064, ZRL20232906) and one European specimen (KM187331) form a distinct, well-supported lineage (BS = 100% and PP = 1.00) nested in this section, providing conclusive phylogenetic evidence for its sectional placement. Meanwhile, the two proposed new species, *L. stillispora* and *L. pallidovelata*, are placed within sect. *Helveolae* and sect. *Lepiota*, respectively, both with strong support (BS = 100% and PP = 1.00) across two datasets. Moreover, these key phylogenetic placements were independently validated by the expanded ITS phylogeny (Suppl. material 6), despite its limited resolution for deep relationships.

### Taxonomic treatment

#### Lepiota

Taxon classificationFungiAgaricalesAgaricaceae

(Pers.) Gray, Nat. Arr. Brit. Pl. 1: 601 (1821)

3B9BB08C-23ED-51DB-98BA-3DE376D5BF48

##### Type species (typ. cons.).

*Agaricus colubrinus* Pers. (= *Lepiota clypeolaria* (Bull.) P. Kumm.).

= *Chamaemyces* Battarra ex Earle, Bull. New York Bot. Gard. 5: 446 (1909).

= *Lepiotella* (E.J. Gilbert) Konrad, Bull. Trimestriel Soc. Mycol. Fr. 50: 26 (1934), non *Lepiotella* J. Rick, Lilloa 2: 251, 1938 (= *Volvolepiota* Singer).

= *Lepiota* subgen. *Lepiotella* E.J. Gilbert, Le Genre Amanita Persoon: 159 (1918).

= *Drosella* Maire, Bull. Soc. Mycol. France 50: 15 (1934), nom. inval. (Art. 38.1(a) and 38.4, ICNafp Madrid Code; Turland et al. 2025).

##### Notes.

The type species of *Chamaemyces*, *C. fracidus*, was recently transferred to *Lepiota* by Li et al. (2025a), but its infrageneric placement remained unresolved. Comprehensive phylogenetic analyses with expanded sampling demonstrate that this species is robustly nested within *L.* sect. *Cristatae*. This finding provides the first sectional placement of this species and supports the merger of *Chamaemyces* (along with its synonyms *Lepiotella* and *L.* subg. *Lepiotella*) into *Lepiota* as established by Li et al. (2025a).

#### Lepiota fracida

Taxon classificationFungiAgaricalesAgaricaceae

(Fr.) R.L. Zhao & J.X. Li, Fungal Divers. 135: 829 (2025).

31FDE963-573A-5F9D-AE5D-C46DB53320A3

Fungal Names: FN 572992

##### Basionym.

*Agaricus fracidus* Fr., Epicr. syst. mycol. (Upsaliae): 25 (1838).

≡ *Armillaria fracida* (Fr.) Gillet, Hyménomycètes: 77 (1874).

≡ *Chamaemyces fracidus* (Fr.) Donk, Beih. Nova Hedwigia 5: 48 (1962).

≡ *Drosella fracida* (Fr.) Singer, Lilloa 22: 446 (1951), nom. inval.

= *Lepiota irrorata* Quél., Compt. Rend. Assoc. Franç. Avancem. Sci. 11: 387 (1883).

= *Lepiotella irrorata* (Quél.) Konrad, Schweiz. Z. Pilzk. 12: 177 (1934).

= *Drosella irrorata* (Quél.) Kühner & Maire, Bull. Soc. Mycol. France 50: 15 (1934), nom. inval.

##### Sectional placement.

*Lepiota* sect. *Cristatae*.

#### Lepiota pallidovelata

Taxon classificationFungiAgaricalesAgaricaceae

X. Li & J. F. Liang sp. nov.

AB78F897-AE31-5610-A4AB-5D80D36AF55A

860343

Fig. 2

##### Holotype.

China • Jilin: Changchun City, Jinyuetan Park, 8 Jul 2004, *J. R. Wang* (holotype, HMJAU3547). GenBank: ITS = MK651643; LSU = MK651684; *rpb*2 = PX913186; mtSSU = MK651732.

**Figure 2.**
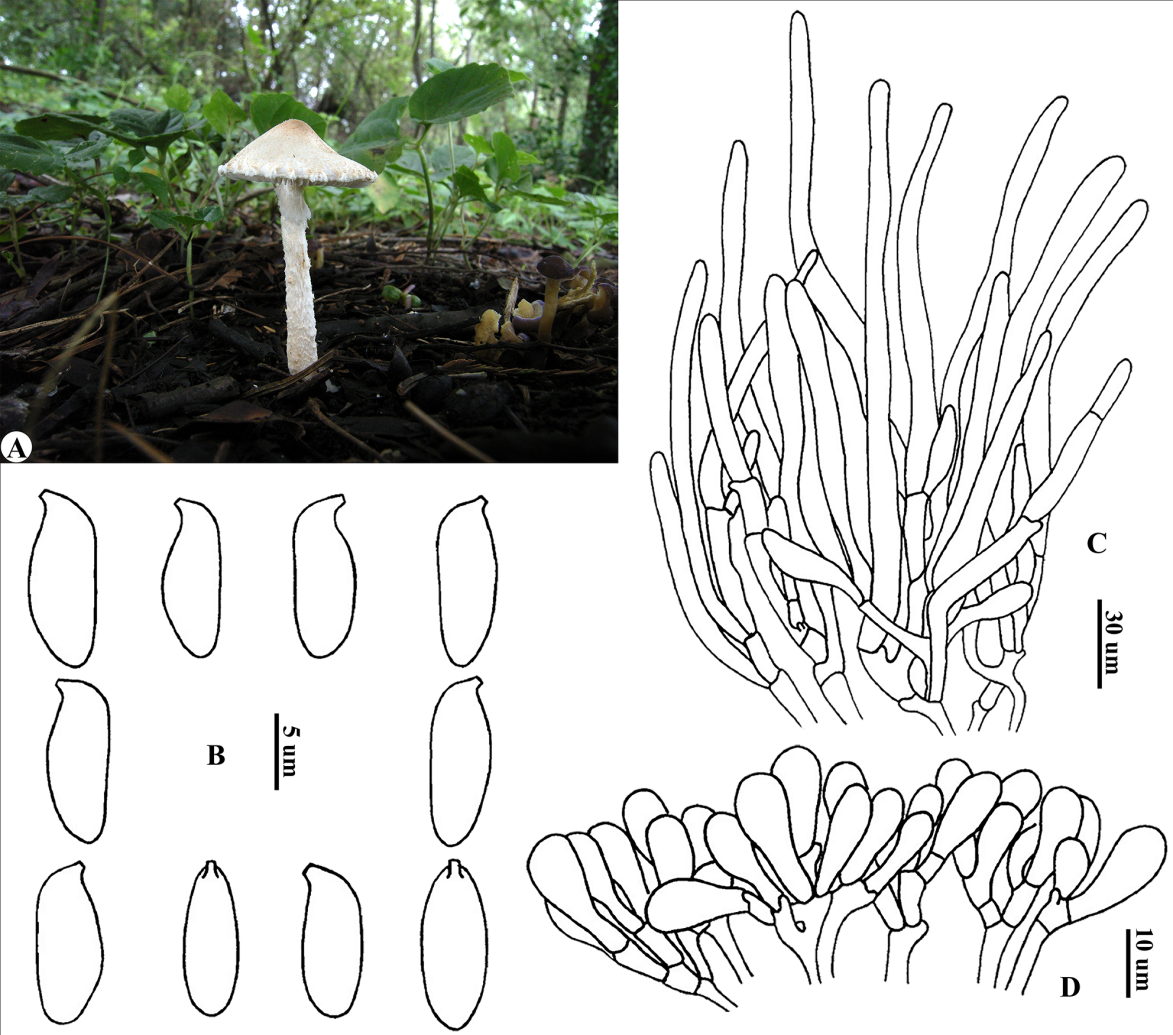
*Lepiota pallidovelata* (holotype, HMJAU3547). **A**. Basidiomata (paratype, HKAS52312); **B**. Basidiospores; **C**. Pileus covering; **D**. Cheilocystidia.

##### Etymology.

pallidovelata (Latin), referring to the distinctive pale (whitish to light brown) squamules on the pileus and the persistent veil remnants at its margin.

##### Diagnosis.

*Lepiota pallidovelata* is characterized by its small basidiomata, pileus with whitish to light brown squamules and veil remnants at the margin, penguin-shaped basidiospores with a suprahilar depression, clavate cheilocystidia, pileus covering a trichoderm composed of long elements (sometimes narrowing towards the apex) and a layer of short elements at their base.

##### Sectional placement.

*Lepiota* sect. *Lepiota*.

##### Description.

***Basidiomata*** small (Fig. 2A). ***Pileus*** 1.5–2.5 cm in diam, initially campanulate, becoming convex, with a blunt, light brown to brown (6B3–6B5) umbo; around the umbo, the surface breaks up into concentric, felted patches to fibrillose squamules on whitish background; squamules becoming smaller and sparser towards the margin, whitish to light brown (6A2–6A4); margin exceeding lamellae, with whitish and fibrillose veil remnants. ***Context*** whitish, thin to slightly thick. ***Lamellae*** L = 40–60, l = 1–2, free, whitish, moderately to densely crowded, ventricose; lamella edge even. ***Stipe*** 2.5–6 × 0.2–0.4 cm, subcylindrical, hollow, narrow to apex, slightly thickened at the base, light brown to brown (5B3–5B4), nearly smooth above the annular zone, lower down with distinct lanate annular zone, below which covered with dense, whitish and floccose squamules. Smell and taste not recorded. Spore print whitish.

***Basidiospores*** (Fig. 2B) [80/4/4] (8.0)9.0–11.5(12.0) × 3.5–5.0(5.5) μm [*Q* = (2.00)2.10–2.88(3.14), *Q*av = 2.51 ± 0.26], penguin-shaped in side view, with suprahilar depression; adaxial side slightly convex; abaxial side nearly straight or sometimes slightly constricted; fusiform in front view; colorless, hyaline, smooth, slightly thick-walled, weakly dextrinoid, congophilous, not metachromatic in cresyl blue; apiculus tiny. ***Basidia*** 23–28 × 8–13 µm, clavate, 4-spored. ***Cheilocystidia*** (Fig. 2D) 12–26 × 5–9 μm, clavate, colorless, hyaline, thin-walled. ***Pleurocystidia*** absent. ***Pileus covering*** (Fig. 2C) a trichoderm composed of subcylindrical, sometimes narrow to apex, terminal elements 58–210 × 6–12 µm rarely bearing septa without clamp connections located in the middle to upper parts, with short and clavate elements at base that contain yellow-brownish intracellular pigment. ***Clamp connections*** present in all tissues.

##### Distribution.

(abbreviation: dist., below). Currently found in Jilin, Yunnan Province, and Beijing City, China.

##### Habitat.

Solitary, saprotrophic on decaying wood and leaf litter. The specimen from Yunnan was found on a decayed branch covered with the vine *Tetrastigma triphyllum* and pine needles, indicating occurrence in a disturbed or forest-edge habitat. Its presence across temperate (Jilin, Beijing) to subtropical (Kunming, ca. 1900 m a.s.l.) mixed forests suggests broad environmental tolerance, potentially including adaptability to secondary environments.

##### Additional specimens examined (paratypes).

China • Jilin: Changchun City, Jinyuetan Park, 13 Jul 2004, *J. R. Wang* (HMJAU3533); • Beijing: Huairou County, Hongluo Temple, old-growth forest, 25 Aug 2000, *T. Bau* (HMJAU1310); • Yunnan: Kunming, Kunming Institute of Botany, 1 Aug 2007, *Yang 4797* (HKAS52312).

##### Nomenclatural note.

The epithet ‘submagnispora’ was provisionally registered in MycoBank (no. 860343). Following critical review, it is here formally amended to ‘pallidovelata’ to more accurately reflect the diagnostic morphological features (i.e., the whitish squamules on the pileus with veil remnants).

##### Notes.

*Lepiota pallidovelata* is distinguished by its tiny basidiomata; blunt and light brown to brown umbonate pileus with whitish to light brown squamules and veil remnants at the margin; penguin-shaped basidiospores without a narrow apex; clavate cheilocystidia; and pileus covering a trichoderm composed of terminal elements, sometimes narrowing toward apex, and basal short elements.

Phylogenetic analysis recovers *L. pallidovelata* as sister to *L. attenuata* Jun F. Liang & Zhu L. Yang with strong support. Both species share the characteristic penguin-shaped basidiospores and a trichodermal pileus covering. They are readily separable morphologically. *Lepiota attenuata* (dist., China: Yunnan) differs in its larger basidiomata, densely covered with brownish yellow to yellowish brown squamules on a whitish background, prominently striate pileus margin, and longer basidiospores that narrow toward the apex (Liang et al. 2011).

Several *Lepiota* species share the penguin-shaped basidiospores and trichodermal pileus covering but can be distinguished from *L. pallidovelata* by distinct morphological features. *Lepiota magnispora* Murrill (dist., North America, Europe, and Asia) differs in having larger basidiomata, longer basidiospores (*Q*av = 3.29), and more diverse cheilocystidia (Liang 2007; Yang et al. 2019). *Lepiota thrombophora* (Berk. & Broome) Sacc. (dist., Sri Lanka; China: Yunnan, Hainan) can be distinguished by reddish brown to dark brown squamules, a striate pileus margin, whitish annulus, longer basidiospores, and a pileus covering rarely with basal short elements (Berkeley and Broome 1871; Liang et al. 2011). *Lepiota ampliocystidia* Jun F. Liang (dist., China: Xizang) differs in its yellowish brown to dark brown squamules on a radially striate pileus margin, lack of veil remnants, whitish annulus, and more elongated basidiospores with a narrowed apex (Liang 2012).

Comparative analysis also reveals distinct morphological features distinguishing *L. pallidovelata* from other *Lepiota* taxa. *Lepiota metulispora* (Berk. & Broome) Sacc. (dist., Sri Lanka, India, and southern China, including Hunan and Hong Kong) differs from *L. pallidovelata* in its appressed, ochraceous buff squamules on a whitish background, striations on the margin of the pileus, stipe with a whitish annulus, longer basidiospores, and gradually narrowing terminal elements in the pileus covering (Liang et al. 2011). Moreover, *L. squamulodiffracta* Justo, Bizzi & Angelini (dist., Dominican Republic) is distinguished from *L. pallidovelata* by its larger basidiomata, brown to orange-brown squamules on the pileus with a striate margin, the absence of veil remnants, and longer basidiospores (Justo et al. 2015).

Additionally, *L. cortinarius* J.E. Lange (dist., Europe, North America, and Asia, including China: Henan, Sichuan, Yunnan, Xizang) can be distinguished from *L. pallidovelata* by its larger basidiomata, dense dark brown squamules, a distinctly cortinate pileus margin, diverse cheilocystidia, and a pileus covering with longer terminal elements (Lange 1915; Liang 2007).

#### Lepiota stillispora

Taxon classificationFungiAgaricalesAgaricaceae

X. Li & J. F. Liang sp. nov.

85A71D52-03E5-523E-8E2B-CD5D6AA8FF5B

860344

Fig. 3

##### Holotype.

China • Guangdong: Dongguan City, Dalingshan Forest Park, 100 m a.s.l., 22°51'47.14"N, 113°45'14.56"E, 8 Jun 2025, *X. Li 056* (holotype, RITF7953). GenBank: ITS = PX423621; LSU = PX423622; *rpb2* = PX913184; mtSSU = PX423648.

**Figure 3.**
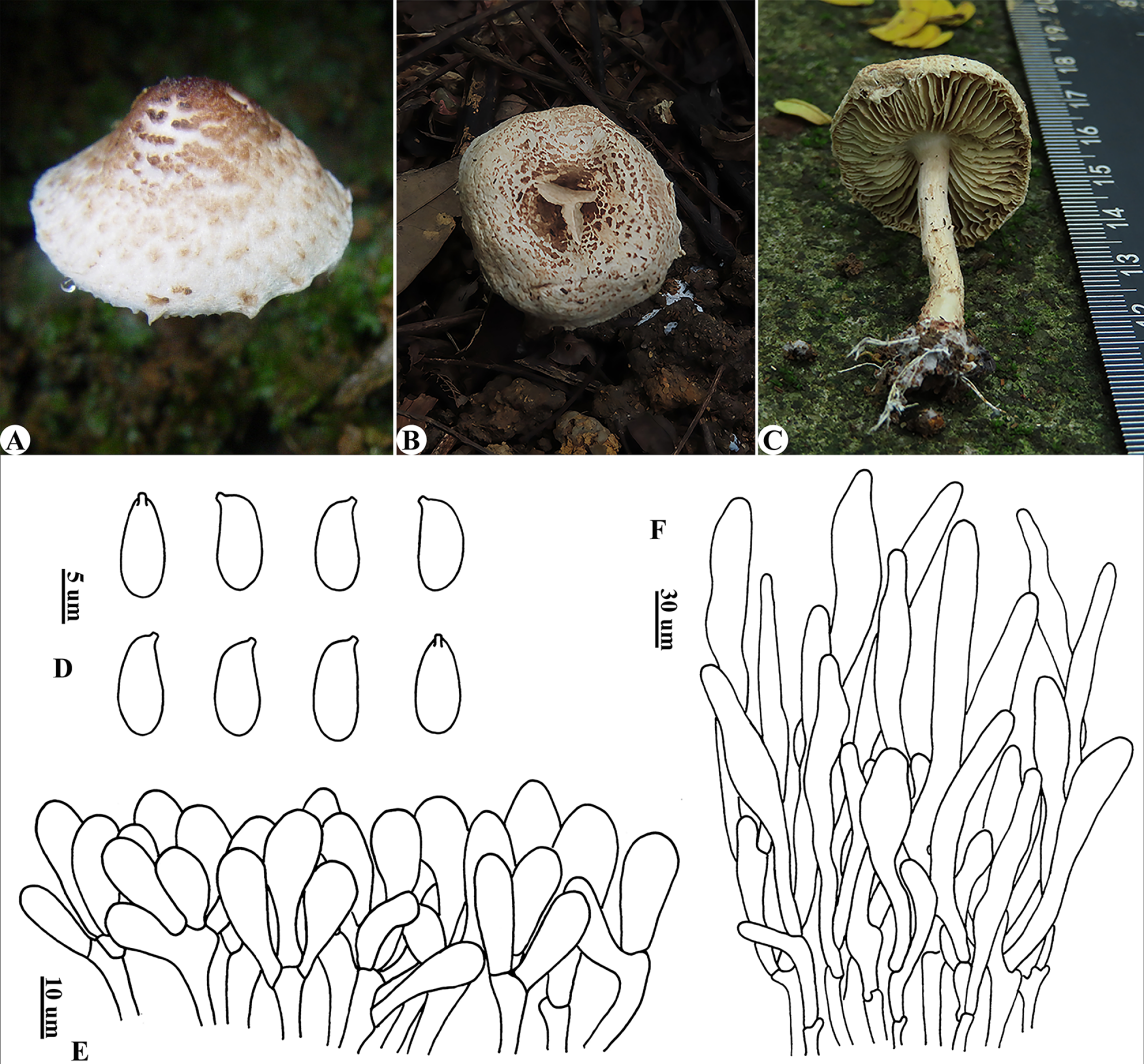
*Lepiota stillispora*. **A**. Basidiomata (HKAS50113); **B, C**. Basidiomata (holotype, RITF7953); **D**. Basidiospores; **E**. Cheilocystidia; **F**. Pileus covering.

##### Etymology.

stillispora (Latin), referring to the droplet-shaped basidiospores.

##### Diagnosis.

*Lepiota stillispora* can be recognized by its dark brownish umbonate and yellowish-brown squamules on the pileus, droplet-shaped (lacrymoid) basidiospores, clavate cheilocystidia, pileus covering a trichoderm composed of terminal elements that are sometimes swollen in the middle and acuminate at the apex, and clamp connections present in all tissues.

##### Sectional placement.

*Lepiota* sect. *Helveolae*.

##### Description.

***Basidiomata*** small (Fig. 3A–C). ***Pileus*** 1.6–3.5 cm in diam, campanulate when young, expanding to plano-convex; with a dark brownish (5C3–5C7) umbo on a dirty-white background; the central region sometimes splitting radially into three lobes at maturity, gradually splitting towards the edge into smaller and yellowish-brown (5B3–5B6) fibrillose squamules; with floccose velar remnants at the margin, sometimes exceeding lamellae. ***Lamellae*** L = 40–60, l = 1–2, moderately crowded, free, whitish, ventricose; edge eroded. ***Stipe*** 2.5–3.5 × 0.2–0.4 cm, subcylindrical, thickening towards base, hollow, whitish and smooth above annulus, with brownish (5A3–5A5) squamules below annulus. ***Annulus*** whitish, ephemeral. Odor not distinct. Taste not recorded. Spore print white.

***Basidiospores*** (Fig. 3D) [40/2/2] 6.5–9.5 × 3.0–5.0 μm [*Q* = (1.51)1.62–2.38(2.47), *Q*av = 2.00 ± 0.38], droplet-shaped or lacrymoid in side view, with suprahilar depression or not; adaxial side usually convex, sometimes substraight, abaxial side often not sunken; bullet-shaped or droplet-shaped in front view; colorless, hyaline, smooth, slightly thick-walled, dextrinoid, light red in Congo red, not metachromatic in cresyl blue; apiculus tiny. ***Basidia*** 18–31 × 7–10 µm, narrowly clavate or subcylindrical, 4-spored. ***Cheilocystidia*** (Fig. 3E) 15–35 × 5–11 µm, clavate, colorless, hyaline, thin-walled, often densely arranged into a sterile lamella edge. ***Pleurocystidia*** absent. ***Pileus covering*** (Fig. 3F) a trichoderm made up of subcylindrical or narrowly clavate, sometimes swollen in the middle part and acuminate on the apex, terminal elements 126–188 × 6–25 µm, lacking a layer of short elements, with yellow-brownish intracellular pigment; base sometimes with pale yellowish-brown to yellowish-brown extracellular pigment. ***Clamp connections*** present in all tissues.

##### Distribution.

Currently known from Guangdong and Yunnan provinces, China.

##### Habitat.

Saprotrophic, solitary on the ground in evergreen monsoon rainforest of limestone mountains in summer.

##### Additional specimens examined.

China • Yunnan: Mengla County, Menglun Town, Lushilin Park, 11 Jul 2006, *J. F. Liang 397* (paratype, HKAS50113).

##### Notes.

The main characteristics of *L. stillispora* are small basidiomata, yellowish brown squamules on the pileus, droplet-shaped or lacrymoid basidiospores, clavate cheilocystidia, and a pileus covering that is a trichoderm composed of terminal elements, sometimes narrowing toward the apex, without short elements at the base.

The basidiospores of *L. stillispora* are similar to those of *L. catenariocystidia* Han C. Wang & Zhu L. Yang (dist., China: Yunnan), but the latter differs in having dark gray and tomentose squamules, cheilocystidia often in chains, and narrower terminal elements of the pileus covering (Wang and Yang 2005).

*Lepiota stillispora* shows morphological differences when compared to some related species. Unlike *L. himalayensis* Khalid & Razaq (dist., Pakistan), which has larger basidiomata, dark brown to blackish squamules on the pileus, and shorter and narrower terminal elements of the pileus covering (Razaq et al. 2012), *L. stillispora* possesses small basidiomata, yellowish brown squamules on the pileus, and longer and thicker terminal elements of the pileus covering. *Lepiota rubella* Bres. (dist., Europe) is characterized by narrower terminal elements of the pileus covering, narrowly clavate cheilocystidia, and smaller basidiospores (Vellinga et al. 1998) than *L. stillispora*. Additionally, *L. stillispora* differs from *L. brunneosquamulosa* Jun F. Liang & Zhu L. Yang (dist., China: Yunnan) in that the latter has smaller basidiospores, no cheilocystidia, and a pileus covering with longer terminal elements that taper toward the apex (Liang et al. 2018).

Furthermore, *L. brunneoincarnata* Chodat & C. Martín (dist., Europe and Asia, including Pakistan and China) and *L. stillispora* exhibit clear morphological differences. The former differs from the latter by bearing larger basidiomata, grayish brown to blackish brown squamules on the pileus, and wider basidiospores (Liang 2007; Yang et al. 2019).

Phylogenetic analysis confirms the close relationship between *L. stillispora* and *L. revelata* (Berk. & Broome) Sacc. Nevertheless, the two species can be readily distinguished by morphological features. *Lepiota revelata* (tropical and subtropical Asia, including Sri Lanka and China) exhibits pale orange lamellae, subcylindrical basidiospores, and very few cheilocystidia (Berkeley and Broome 1871; Yang et al. 2019).

## Discussion

### Phylogenetic framework of *Lepiota* and the position of *L. fracida*

The mtSSU-based phylogeny (Fig. 1), based on the same sequence matrix (ITS, LSU, *rpb*2, mtSSU) as that used by Sarawi et al. (2025a), provides strong support for their recently established seven-section classification of *Lepiota*, which confirms the overall stability of this infrageneric framework. Key relationships, such as the clade formed by *L.* sect. *Cristatae*, *Helveolae*, and *Stenosporae*, as well as the basal position of sect. *Eriophorae* are recovered with high statistical support, reinforcing their validity. However, the topology places *L. angusticystidiata* within the clade (BS = 90% and PP = 0.63) containing sect. *Lilaceae* and sect. *Lepiota*. This contrasts with that of Sarawi et al. (2025a), who recovered sect. *Lilaceae* and *Lepiota* as a joint clade without the inclusion of *L. angusticystidiata*. This topological difference likely results from the more comprehensive dataset for *L. angusticystidiata*, which includes all four loci sequenced from both holotype and paratype specimens, whereas Sarawi et al. (2025a) only analyzed the holotype without the *rpb*2 locus. Consequently, this analysis robustly resolves *L. angusticystidiata* as a distinct and well-supported lineage (BS = 100% and PP = 1.00) that is sister to sect. *Lepiota* (BS = 100% and PP = 1.00), thereby clarifying its phylogenetic position and underscoring the necessity of comprehensive data.

However, the sequence matrix of the mtSSU-based phylogeny is different from that of the phylogeny (ITS, LSU, *rpb*2, *tef*1-α) of Li et al. (2025a). In the *Lepiota* clade within their phylogenetic tree, key relationships lacked statistical support, major clades remained unresolved, and the resulting topology differed significantly from those recovered in previous studies (Hou and Ge 2020; Ge et al. 2021) using the same matrix. In view of these observations, the *tef*1-α-based phylogeny (Suppl. material 5) was reconstructed using the same four-gene combination with 83 selected taxa to verify the consistency of results derived from this matrix. However, only 29 available *tef*1-α (vs. 83 ITS, 81 LSU, and 82 *rpb*2) sequences met the sampling criterion of requiring at least three loci per taxon for robust phylogenetic inference. This highlights a practical challenge in *Lepiota* phylogeny posed by the limited sequence overlap between the two widely used gene combinations (one including mtSSU and the other *tef*1-α) for the same specimens, which makes combined analyses difficult without compromising taxon sampling. This finding further supports the value of including mtSSU in the mtSSU-based phylogeny (80 ITS, 80 LSU, 73 *rpb*2, and 70 mtSSU), which provides high data completeness with minimal missing data. This is in sharp contrast to the persistent shortage of available *tef*1-α sequences in critical taxa, a limitation that is also present in the *tef*1-α-based and previous (Hou and Ge 2020; Ge et al. 2021; Li et al. 2025a) phylogenies. If the issue of missing data is excluded, the *tef*1-α-based phylogeny (Suppl. material 5) is consistent with previous ones (Hou and Ge 2020; Ge et al. 2021) and the mtSSU-based phylogeny in several key relationships. The topology recovers the clade formed by sect. *Cristatae* (BS = 81% and PP = 0.95), *Helveolae* (BS = 98% and PP = 0.96), and *Stenosporae* (BS = 99% and PP = 1.00) with high statistical support, as well as the basal position of sect. *Eriophorae* (BS = 100% and PP = 1.00). Two samples of *L. angusticystidiata* are also resolved as a distinct, fully supported lineage (BS = 100% and PP = 1.00) that is sister to sect. *Lepiota* (BS = 99% and PP = 1.00), and together they also form a well-supported monophyletic clade (BS = 98% and PP = 1.00). The only difference among them lies in the unstable phylogenetic positions of sect. *Fuscovinaceae* and sect. *Lilaceae*, because all analyses show that these two sections receive weak or no support, indicating that additional data may be required to further clarify their delimitations.

Therefore, within this largely clarified phylogenetic framework mentioned above, this study resolves the sectional placement of *L. fracida*. By incorporating sequences from Asian and European samples, this analysis robustly nests *L. fracida* within sect. *Cristatae* with full support (BS = 100% and PP = 1.00). This placement is stable in the mtSSU-based phylogeny (Fig. 1), *tef*1-α-based phylogeny (Suppl. material 5), and ITS phylogeny (Suppl. material 6), providing conclusive phylogenetic evidence.

### Morphological re-evaluation supports the prior merger of *Chamaemyces* into *Lepiota*

The robust phylogenetic placement of *L. fracida* within sect. *Cristatae* is corroborated by a detailed re-evaluation of its morphology. This species aligns precisely with the sectional diagnosis emended by Sarawi et al. (2025a). Key diagnostic features consistent with this placement include ellipsoid basidiospores and the presence of clamp connections.

#### Basidiospores

Section *Cristatae* is defined as encompassing species with ellipsoid, sometimes spurred, mainly non-dextrinoid but sometimes dextrinoid basidiospores (Sarawi et al. 2025a). *Lepiota fracida* produces ellipsoid, non-dextrinoid spores (Yang et al. 2019; Vellinga 2001), a state that fits well with the circumscription of this section, but its spore wall is metachromatic in cresyl blue (Vellinga 2001; Yang et al. 2019). Among *Lepiota* species, spores are typically either non-metachromatic in cresyl blue or show only a pink inner wall (Vellinga 2001). However, this reaction pattern is not consistent across *Lepiota*, even at the sectional level. Within sect. *Cristatae* itself, for instance, both metachromatic in cresyl blue (e.g., *L. cristatanea* Jun F. Liang & Zhu L. Yang, *L. atrobrunneodisca* L. Fan, L. Xia & N. Mao, and *L. coloratipes* Vizzini, Jun F. Liang, Jančovič. & Zhu L. Yang) and non-metachromatic (e.g., *L. sanguineofracta* Vizzini and *L. condylospora* Sysouphanthong, K.D. Hyde & Vellinga) species occur. Thus, the metachromatic reaction of *L. fracida* falls within the observed range of variation for sect. *Cristatae* and does not argue against its placement in this section.

#### Clamp connections

The presence of abundant clamp connections in all tissues is characteristic of sect. *Cristatae* (Sarawi et al. 2025a) and is explicitly confirmed for *L. fracida* in previous studies (Singer 1986; Vellinga 2001, 2003; Yang et al. 2019), further reinforcing its placement within *Lepiota*.

While the above features support the inclusion of *L. fracida* in *Lepiota* sect. *Cristatae*, several other characters have historically been emphasized to justify the generic distinction of *Chamaemyces*, i.e., the gelatinized pileus covering, stipe droplet exudation, the presence of pleurocystidia, and the unique developmental mode. All of these characters are shown herein to lack taxonomic utility for generic delimitation.

#### Pileus covering

This hymeniform pileus covering is consistently and precisely reported for *L. fracida* across several major monographs (Singer 1986; Vellinga 2001; Yang et al. 2019), where it is described as a hymeniderm composed of clavate to broadly clavate cells. However, the hymeniform pileus covering is present not only in sect. *Cristatae*, but also in sect. *Lilaceae* (Sarawi et al. 2025a). Section *Lilaceae* is defined by a vivid orange-brown or dark violet-brown pileus (further divided into two subsections by pileus color, i.e., subsect. *Paralilaceae* and *Lilaceae*), a hymeniform pileus covering, and ellipsoid basidiospores that are either dextrinoid and longer than 5 μm or non-dextrinoid and rather small (with a dark underside to the annulus), with no disc-concentrated pigmentation and pale-marginal scattered scales on the pileus. In contrast, sect. *Cristatae* is characterized by an orange to dark brown pileus with disc-concentrated pigmentation and scattered colored scales on a pale background toward the margin; a hymeniform pileus covering (sometimes with additional spherical agglutinated cells); and ellipsoid (rarely spurred) basidiospores, and it never possesses the combined pileus, basidiospore, and annulus characters typical of sect. *Lilaceae*. *Lepiota fracida* has an ochre-yellow to creamy pinkish pileus at the center to very pale yellow at the margin, the absence of an annulus with a dark underside, a hymeniform pileus covering, and ellipsoid and non-dextrinoid basidiospores (Vellinga 2001; Didukh et al. 2004; Yang et al. 2019). This suite of morphological characters is consistent with the circumscription of sect. *Cristatae* and lacks the defining combined traits of sect. *Lilaceae*, thus firmly supporting its placement in sect. *Cristatae*.

Furthermore, the pileus of *L. fracida* has been reported to be slightly viscid when moist in some studies because of the presence of a gelatinous layer (Vellinga 2001; Yang et al. 2019). However, populations of this species in Israel lack a gelatinized pileus and are described as almost dry (Didukh et al. 2004). This distinct intraspecific variation, as definitively evidenced by Didukh et al. (2004), clearly indicates that pileus viscidity is an unstable and non-diagnostic character for this species and does not conflict with its placement in sect. *Cristatae*.

#### Stipe droplets

Exudation of small droplets on the stipe is observed in *L. fracida* (Vellinga 2001; Didukh et al. 2004; Kibby 2015; Yang et al. 2019; Mattock and Kibby 2021), yet this feature is not a diagnostic character for the genus *Chamaemyces* since its congeneric species *C. carmelensis* M. Didukh & Wasser entirely lacks the exudation of droplets (Didukh et al. 2004). Stipe droplets also occur in some species of *Leucocoprinus* Pat., (1888), including *Lc. tangerinus* (Y. Yuan & Jun F. Liang) Kun L. Yang, Jia Y. Lin & Zhu L. Yang, which produces droplets on the lower part of the stipe (Yuan et al. 2014). However, this character is relatively common across the genus, and it remains unknown whether such species form a monophyletic group or whether stipe droplets possess phylogenetic significance. This consistency across genera indicates that stipe droplets are characteristic of individual species rather than entire genera, demonstrating that this character is not taxonomically useful for generic delimitation. Thus, the presence of stipe droplets in *L. fracida* does not justify its separation from *Lepiota*.

#### Pleurocystidia

*Lepiota fracida* is described as having abundant pleurocystidia (Vellinga 2001; Yang et al. 2019), a feature historically considered a diagnostic characteristic of the genus *Chamaemyces*. Furthermore, although pleurocystidia are far from universal within the genus *Lepiota*, their presence has been confirmed and documented in some species (e.g., in *L. pleurocystidiata* Sysouph., Thongkl. & K.D. Hyde). Therefore, this character represents a specific morphological state within *L. fracida* but does not constitute the autapomorphy of *Chamaemyces* that would conflict with its placement.

#### Developmental mode

The basidioma development of *L. fracida* is characterized as monovelangiocarpic and stipitocarpic, and the latter has also been documented for *L. cristata* (Bolton) P. Kumm., the type of sect. *Cristatae* (Reijnders 1975). Stipitocarpic development is therefore not a unique feature of *L. fracida* and poses no morphological conflict to its placement within sect. *Cristatae*. This leaves monovelangiocarpic development as the sole trait that was once considered the cornerstone for maintaining *Chamaemyces* as a separate genus (Vellinga 2001, 2003; Yang et al. 2019). However, the phylogenetic results necessitate a re-evaluation of this character’s diagnostic weight. Two key lines of evidence argue against its generic-level taxonomic significance. First, and most critically, the genus *Lepiota* itself exhibits considerable developmental plasticity in stipe-pileus ontogeny, with forms ranging from stipitocarpic (*L. cristata*) to pileostipitocarpic (other *Lepiota* species, e.g., *L. clypeolaria*). This plasticity is further reflected in the interspecific divergence in the morphology and structure of the universal veil within the genus (Reijnders 1975). *Lepiota cristata* possesses scanty lipsanenchyma and no external radiating hyphae in the universal veil, while *L. clypeolaria* bears a universal veil with radiating hyphae on the outside that cover both the pileus and the upper part of the stipe. An even more distinct morphological variation is displayed by *L. ignipes* Locq. ex Bon (= *L. castanea*), which has radiating hyphae of the universal veil restricted to the inner side, a spatial distribution that is fully opposite to that of other congeneric species. This pronounced variation in universal veil traits, together with the ontogenetic differences of stipe-pileus development, directly indicates that developmental patterns related to basidioma construction are inherently labile. In addition, the fully sequestrate species (Ge and Smith 2013; Lebel and Vellinga 2013; Vidal et al. 2015; Paz and Lavoise 2024; Li et al. 2025b; Sarawi et al. 2025a) occur in sect. *Lepiota*. This form represents a morphologically specialized shift from the typical agaricoid form, and this difference is far more pronounced than the variations in velar configuration seen in *L. fracida*. Therefore, if such a pronounced morphological shift can have occurred multiple times within a single section of *Lepiota*, the subtle developmental differences of *L. fracida* appear insufficient to justify its generic segregation from *Lepiota*. Second, and conclusively, the robust phylogenetic position of *L. fracida* within sect. *Cristatae* directly contradicts a classification based solely on its development pattern. This demonstrates that these historically emphasized developmental characters have limited phylogenetic signal and are better viewed as homoplastic characters that evolved independently in specific evolutionary contexts, as foreseen by Reijnders (1975). Such homoplastic developmental characters lack taxonomic value for generic delimitation and therefore cannot serve as reliable diagnostic characteristics for distinguishing genera, only as species-specific features.

In the framework of modern systematics, where classification must reflect monophyletic groups established on robust molecular evidence, those characters that are either non-diagnostic or potentially homoplastic, such as developmental modes, must be subordinate to well-supported phylogenetic relationships and to conserved, definitive morphological synapomorphies. Consequently, for *L. fracida*, the re-evaluated morphological characters not only align with but also substantiate its phylogenetic placement, providing further support for the prior merger of *Chamaemyces* into *Lepiota*, proposed by Li et al. (2025a). It should be noted that this conclusion is based on data from the type species of *Chamaemyces*. The phylogenetic placement of other species historically assigned to this genus remains unresolved and requires further study to determine whether they also belong to *Lepiota* or represent a distinct lineage.

### Safety considerations for the new species

An important consideration arising from this study pertains to the chemical profile and potential toxicity of the newly described *L. stillispora*. This species is placed within *L.* sect. *Helveolae*, a group known to contain species (e.g., *L. brunneoincarnata*) producing deadly amatoxins (Sgambelluri et al. 2014; Sarawi et al. 2022, 2025b). To date, no chemical data are available for *L. stillispora*. Therefore, in the interest of public safety, consumption is strongly discouraged until its edibility has been rigorously established through future chemical studies.

## Supplementary Material

XML Treatment for Lepiota

XML Treatment for Lepiota fracida

XML Treatment for Lepiota pallidovelata

XML Treatment for Lepiota stillispora

## Additional information

### Artificial Intelligence (AI) use

The authors accept full responsibility for the content of the manuscript, including the disclosure of any use of AI.

No AI tools were used in the preparation of this manuscript.

### Author ORCIDs

X. Li https://orcid.org/0000-0001-7146-0125

B. Chen https://orcid.org/0000-0001-8459-3752

M. An https://orcid.org/0009-0001-6170-5886

J. Liang https://orcid.org/0000-0002-0433-2577

### Data availability

All data supporting this study are available within the article and its supplementary materials. The newly generated DNA sequences have been deposited in GenBank (accession numbers: mtSSU-based and ITS phylogenies in Suppl. material 1; *tef*1-α-based phylogeny in Suppl. material 5). The mtSSU-based phylogeny alignments and resulting phylogenetic tree files are available as Suppl. materials 2–4. The *tef*1-α-based phylogeny and the ITS phylogeny tree are available as Suppl. materials 5, 6, respectively.

## Supplementary materials

Supplementary material 1 Sampled species Data type doc Explanation note A list of the vouchers, location and GenBank accession numbers for the sampled species in this study. https://binary.pensoft.net/file/1659825This dataset is made available under the Open Database License (http://opendatacommons.org/licenses/odbl/1.0/). The Open Database License (ODbL) is a license agreement intended to allow users to freely share, modify, and use this Dataset while maintaining this same freedom for others, provided that the original source and author(s) are credited. Xing Li

Supplementary material 2 Phylogeny alignment Data type fas Explanation note Alignment of concatenated dataset (ITS, LSU, rpb2, mtSSU). https://binary.pensoft.net/file/1659826This dataset is made available under the Open Database License (http://opendatacommons.org/licenses/odbl/1.0/). The Open Database License (ODbL) is a license agreement intended to allow users to freely share, modify, and use this Dataset while maintaining this same freedom for others, provided that the original source and author(s) are credited. Xing Li

Supplementary material 3 Treefile Data type treefile Explanation note Treefile for concatenated dataset (ITS, LSU, rpb2, mtSSU). https://binary.pensoft.net/file/1659827This dataset is made available under the Open Database License (http://opendatacommons.org/licenses/odbl/1.0/). The Open Database License (ODbL) is a license agreement intended to allow users to freely share, modify, and use this Dataset while maintaining this same freedom for others, provided that the original source and author(s) are credited. Xing Li

Supplementary material 4 Annotation for treefile Data type txt https://binary.pensoft.net/file/1659828This dataset is made available under the Open Database License (http://opendatacommons.org/licenses/odbl/1.0/). The Open Database License (ODbL) is a license agreement intended to allow users to freely share, modify, and use this Dataset while maintaining this same freedom for others, provided that the original source and author(s) are credited. Xing Li

Supplementary material 5 Phylogenetic tree Data type doc https://binary.pensoft.net/file/1659829This dataset is made available under the Open Database License (http://opendatacommons.org/licenses/odbl/1.0/). The Open Database License (ODbL) is a license agreement intended to allow users to freely share, modify, and use this Dataset while maintaining this same freedom for others, provided that the original source and author(s) are credited. Xing Li

Supplementary material 6 Phylogenetic tree Data type jpg Explanation note Phylogenetic tree based on ITS sequence. https://binary.pensoft.net/file/1659830This dataset is made available under the Open Database License (http://opendatacommons.org/licenses/odbl/1.0/). The Open Database License (ODbL) is a license agreement intended to allow users to freely share, modify, and use this Dataset while maintaining this same freedom for others, provided that the original source and author(s) are credited. Xing Li
