# Supplementary material for: Studies in Lepiota (Agaricales, Verrucosporaceae): supporting the merger of Chamaemyces into Lepiota and proposing two new species
Source: MycoKeys. 2026 May 28;133:103–25. doi: 10.3897/mycokeys.133.186351 (PMC13237568; doi:10.3897/mycokeys.133.186351)
Supplement: Supplementary material 1 — Sampled species [file mycokeys-133-103-s001.doc]

**Supplementary Table 1.** A list of the vouchers, location and GenBank accession numbers for the sampled species in this study.

| **Part A: Four-locus (ITS, LSU, *rpb2*, mtSSU) dataset (N=80)** | | | | | | | | |
| --- | --- | --- | --- | --- | --- | --- | --- | --- |
| **No.** | **Taxon** | **Voucher** | **Location** | **ITS** | **LSU** | ***rpb2*** | **mtSSU** | **References** |
| 1 | *L. alba* | HKAS 90371 | China, Xinjiang | MN810115 | MN810075 | MN820946 |  | Hou and Ge 2020 |
| 2 | *L. albogranulosa* | SeSa342 | Benin | PP594605 | PP594728 | PP841237 | PQ152809 | Sarawi et al. 2025a, 2025b |
| 3 | *L. angusticystidiata* | HKAS 50064 (HT) | China, Yunnan | KP177192 | KP177198 | **MK705838** | KP177202 | Liang et al. 2018 |
| 4 | *L. angusticystidiata* | RITF569 (PT) | China, Yunnan | KP177194 | KP177199 | **MK705839** | **MK690175** | Liang et al. 2018 |
| 5 | *L. apatelia* | BH20210810 | Germany | PP594619 | PP594746 | PP841157 | PQ152823 | Sarawi et al. 2025a, 2025b |
| 6 | *L. atrobrunneodisca* | HSA 115 (HT) | China | OP724226 | OP724232 |  | OP724234 | Mao et al. 2023 |
| 7 | *L. attenuata* | HKAS50110 (HT) | China, Yunnan | EU681776 | GU199354 | MK705816 | EU681826 | Liang et al. 2011 |
| 8 | *L. aurantiicolor* | SeSa176 (HT) | Benin | PP594563 | PP594681 | PP841188 | PQ152789 | Sarawi et al. 2025a, 2025b |
| 9 | *L. baiyunensis* | B22052705 (HT) | China | NR_198415 | OQ547188 |  | OQ547190 | Liang et al. 2023 |
| 10 | *L. beninensis* | FC-21-201 (HT) | Benin | PP594630 | PP594755 | PP841161 | PQ152830 | Sarawi et al. 2025a, 2025b |
| 11 | *L. boudieri* | SeSa13 | Austria | OL527685 | PP594636 | PP841186 | PQ152758 | Sarawi et al. 2022, 2025a, 2025b |
| 12 | *L. brunneoincarnata* | SeSa54 | Germany | OL527694 | PP594648 | PP841256 | PQ152765 | Sarawi et al. 2022, 2025a, 2025b |
| 13 | *L. brunneolilacea* | RITF617 | USA, Arkansas | MK651609 | MK651664 | **MK705806** | MK651700 | Li X et al. 2025 |
| 14 | *L. brunneoolivacea* | SeSa214 (HT) | Benin | PP594574 | PP594693 | PP841201 | PQ152794 | Sarawi et al. 2025a, 2025b |
| 15 | *L. brunneophora* | RITF541 (PT) | China | MK651649 | MK685364 | **MK705830** | MK651736 | Li X et al. 2025 |
| 16 | *L. brunneosquamulosa* | HKAS 50142 (HT) | China, Yunnan | NR_169900 | NG_073598 |  | KP177203 | Liang et al. 2018 |
| 17 | *L. carinii* | HB291004 | Germany | PQ152700 | PQ152728 | PQ202731 | PQ152754 | Sarawi et al. 2025a |
| 18 | *L. castanea* | SeSa39 | Germany | PP594529 | PP594641 | PP841249 | PQ152761 | Sarawi et al. 2025a, 2025b |
| 19 | *L. clypeolaria* | HKAS46074 | USA, Arkansas | MK651621 | MK651670 | **MK705818** | EU681814 | Li X et al. 2025 |
| 20 | *L. clypeolaria* | HKAS52854 | USA, Arkansas | MK651622 | MK685363 | **MK705819** | MK651713 | Li X et al. 2025 |
| 21 | *L. cortinarius* | HKAS 46095 | China, Tibet | EU416306 | EU416307 | **MK705820** | EU681823 | Liang et al. 2010 |
| 22 | *L. cristata* | SeSa70 | Austria | PP594542 | PP594658 | PP841265 | PQ152771 | Sarawi et al. 2025a, 2025b |
| 23 | *L. cristatanea* | HKAS50021 (HT) | China, Yunnan | EU081952 | **MK651659** | **MK705836** | EU082003 | Liang et al. 2009 |

Supplementary Table 1. (Continued)

| **No.** | **Taxon** | **Voucher** | **Location** | **ITS** | **LSU** | ***rpb2*** | **mtSSU** | **References** |
| --- | --- | --- | --- | --- | --- | --- | --- | --- |
| 24 | *L. echinacea* | HB290998 | Germany | PQ152702 | PQ152729 | PQ202733 | PQ152750 | Sarawi et al. 2025a |
| 25 | *L. echinobispora* | SeSa115 (HT) | Benin | PP594560 | PP594678 | PP841184 | PQ152787 | Sarawi et al. 2025a, 2025b |
| 26 | *L. elaiophylla* | SeSa363 | Germany | PP594611 | PP594734 | PP841243 | PQ152813 | Sarawi et al. 2025b |
| 27 | *L. erminea* | SeSa98 | Germany | PP594552 | PP594670 | PP841276 | PQ152782 | Sarawi et al. 2025a, 2025b |
| 28 | *L. farinolens* | PR20210907 | Germany | PP594618 | PP594745 | PP841167 | PQ152822 | Sarawi et al. 2025a, 2025b |
| 29 | *L. felina* | SeSa57 | Germany | PP594535 | PP594651 | PP841258 | PQ152767 | Sarawi et al. 2025a, 2025b |
| 30 | *L. felina* | SeSa7 | Germany | PP594523 | PP594633 | PP841264 | PQ152736 | Sarawi et al. 2025a, 2025b |
| 31 | *L. flavonigrescens* | SeSa305 (HT) | Benin | PP594596 | PP594717 | PP841226 | PQ152805 | Sarawi et al. 2025a, 2025b |
| 32 | *L. fracida* | KM187331 | United Kingdom, Wales | PP763181 | PP763097 | PP829104 |  | Pepijn et al. 2024 |
| 33 | *L. fracida* | ZRL20232064 | China | PV607755 | PV607719 | PV614752 |  | Li J et al. 2025 |
| 34 | *L. fracida* | ZRL20232096 | China | PV607756 | PV607720 | PV614753 |  | Li J et al. 2025 |
| 35 | *L. fuscovinacea* | SeSa8 | Germany | PP594524 | PP594634 | PP841268 | PQ152738 | Sarawi et al. 2025a, 2025b |
| 36 | *L. geocarpa* | UTC00143916 | USA, Utah | HQ020412 | EU130550 | MN820945 |  | Kropp et al. 2012 |
| 37 | *L. geophana* | UTC00253060 (HT) | USA | HQ020411 | HQ020421 | MN820944 |  | Kropp et al. 2012 |
| 38 | *L. grangei* | KaiR1768 | Germany | PP594624 | PP594638 | PP841166 | PQ152826 | Sarawi et al. 2025a, 2025b |
| 39 | *L. griseovirens* | SeSa90 | Germany | PP594547 | PP594664 | PP841271 | PQ152776 | Sarawi et al. 2025a, 2025b |
| 40 | *L. ignivolvata* | SeSa97 | Germany | PP594551 | PP594669 | PP841275 | PQ152781 | Sarawi et al. 2025a, 2025b |
| 41 | *L. jacobi* | SeSa76 | Germany | PP594543 | PP594659 | PP841266 | PQ152772 | Sarawi et al. 2025a, 2025b |
| 42 | *L. kuehneriana* | HKAS45723 | China, Tibet | GU199360 | GU199358 | **MK705821** | EU681825 | Liang et al. 2011 |
| 43 | *L. lilacea* | SeSa367 | Germany | PP594612 | PP594735 | PP841244 | PQ152814 | Sarawi et al. 2025a, 2025b |
| 44 | *L. lilaceostriata* | SeSa280 (HT) | Benin | PP594588 | PP594709 | PP841217 | PQ152800 | Sarawi et al. 2025a, 2025b |
| 45 | *L. longisterigmata* | SeSa179 (HT) | Benin | PP594565 | PP594683 | PP841190 | PQ152790 | Sarawi et al. 2025a, 2025b |
| 46 | *L. magnispora* | SeSa68 | Austria | PP594541 | PP594657 | PP841263 | PQ152770 | Sarawi et al. 2025a, 2025b |
| 47 | *L. mengei* | UTC00253524 (PT) | USA, Utah | MN810131 | MN810082 | MN820942 |  | Hou and Ge 2020 |
| 48 | *L. metulispora* | HMGID25584 | China | MK651632 | MK651674 |  | MK651721 | Li X et al. 2025 |
| 49 | *L. minutisterigmata* | SeSa343 (HT) |  | PP594606 | PP594729 | PP841238 | PQ152810 | Sarawi et al. 2025a, 2025b |

Supplementary Table 1. (Continued)

| **No.** | **Taxon** | **Voucher** | **Location** | **ITS** | **LSU** | ***rpb2*** | **mtSSU** | **References** |
| --- | --- | --- | --- | --- | --- | --- | --- | --- |
| 50 | *L. minutoflava* | SeSa283 (PT) | Benin | PP594590 | PP594711 | PP841219 | PQ152802 | Sarawi et al. 2025a, 2025b |
| 51 | *L. neophana* | RITF2402 | China | **MK651599** | **MK651661** | **MK705837** | **MK651694** | **This study** |
| 52 | *L. ochraceofulva* | SeSa100 | Germany | PP594554 | PP594672 | PP841178 | PQ152783 | Sarawi et al. 2025a, 2025b |
| 53 | *L. ochraceosquamea* | HKAS45559 (HT) | USA, Arkansas | MK651634 | MK651676 |  | MK651724 | Li X et al. 2025 |
| 54 | *L. oreadiformis* | SeSa96 | Germany | PP594550 | PP594668 | PP841274 | PQ152780 | Sarawi et al. 2025a, 2025b |
| 55 | *L. pilodes* | SeSa379 | Germany | PP594614 | PP594736 | PP841247 | PQ152816 | Sarawi et al. 2025a, 2025b |
| 56 | *L. psalion* | FC20103101 | Germany | PP594616 | PP594743 | PP841160 | PQ152820 | Sarawi et al. 2025a, 2025b |
| 57 | *L. pseudolilacea* | HKAS8288 | China, Jilin | EU416304 | EU416305 | **MK705808** | EU681808 | Liang et al. 2010 |
| 58 | *L. pseudovenenosa* | SeSa302 (HT) |  | PP594593 | PP594714 | PP841223 | PQ152804 | Sarawi et al. 2025a, 2025b |
| 59 | *L. punaensis* | HAW-F-00256 (HT) | USA, Hawai | NR_173815 | NG_153999 | OM215203 |  | Stallman et al. 2020; |
| 60 | ***L. revelata*** | **HKAS50111** | **USA, Arkansas** | **MK651616** | **MK651669** | **MK705813** | **PX105040** | **This study** |
| 61 | *L. rubella* | SeSa87 | Germany | PP594544 | PP594661 | PP841269 | PQ152773 | Sarawi et al. 2025a, 2025b |
| 62 | *L. rubrobrunnea* | JKS118 ITS / HAW-F-00254 LSU & rpb2 | USA, Hawaii | MK412583 | OM203354 | OM215204 |  | Stallman et al. 2020 |
| 63 | *L. rufobrunnea* | HKAS52674 | USA, Arkansas | MK651626 | MK685362 |  | MK651715 | Li X et al. 2025 |
| 64 | *L. spheniscispora* | ecv2556 (HT) ITS/ ecv2438 LSU / ecv2817 rpb2 | USA, California | NR_119448 | AY176404 | HM488813 |  | Vellinga 2001a; Vellinga et al. 2011 |
| 65 | *L. spiculata* | RC-MART-10-072 | France | PP594628 | PP594753 | PP841168 | PQ152828 | Sarawi et al. 2025a, 2025b |
| 66 | ***L. stillispora*** | **LX056 (HT)** | **China, Guangdong** | **PX423621** | **PX423622** | **PX913184** | **PX423648** | **This study** |
| 67 | ***L. stillispora*** | **HKAS50113 (PT)** | **China, Yunnan** | **PX105037** | **PX105041** | **PX913185** | **PX105039** | **This study** |
| 68 | *L. subgracilis* | HKAS 5802 | China, Jilin | EU416290 | EU416291 | **MK705815** | EU681811 | Liang et al. 2010 |
| 69 | *L. subincarnata* | SeSa49 | Germany | PP594533 | PP594646 | PP841254 | PQ152763 | Sarawi et al. 2025a, 2025b |
| 70 | ***L. pallidovelata*** | **HMJAU1310 (PT)** | **USA, Arkansas** | **MK651642** | **MK651683** |  | **MK651731** | **This study** |
| 71 | ***L. pallidovelata*** | **HMJAU3547 (HT)** | **China** | **MK651643** | **MK651684** | **PX913186** | **MK651732** | **This study** |
| 72 | ***L. pallidovelata*** | **HMJAU3533 (PT)** | **China** | **MK651644** | **MK651685** | **PX913187** | **MK651733** | **This study** |
| 73 | ***L. pallidovelata*** | **HKAS52312 (PT)** | **China, Yunnan** | **MK651645** | **MK685369** |  | **MK651734** | **This study** |

Supplementary Table 1. (Continued)

| **No.** | **Taxon** | | **Voucher** | | **Location** | | **ITS** | **LSU** | | ***rpb2*** | | **mtSSU** | **References** | | |
| --- | --- | --- | --- | --- | --- | --- | --- | --- | --- | --- | --- | --- | --- | --- | --- |
| 74 | *L. thrombophora* | | HKAS41003 | | China, Hainan | | EU681780 | MK651687 | | **MK705829** | | EU681813 | Liang et al. 2011 | | |
| 75 | *L. tomentella* | | SeSa58 | | Germany | | PP594536 | PP594652 | | PP841259 | | PQ152768 | Sarawi et al. 2025a, 2025b | | |
| 76 | *L. tyrianthina* | | SeSa213 (HT) | | Benin | | PP594573 | PP594692 | | PP841200 | | PQ152793 | Sarawi et al. 2025a, 2025b | | |
| 77 | *L. woehnertii* | | SeSa256 (HT) | | Benin | | PP594583 | PP594704 | | PP841212 | | PQ152798 | Sarawi et al. 2025a, 2025b | | |
| 78 | *Echinoderma asperum* | | SeSa62 | | Germany | | PP594540 | PP594656 | | PP841262 | | PQ152769 | Sarawi et al. 2025a, 2025b | | |
| 79 | *Echinoderma asperum* | | TL20041019 | | Germany | | PQ152688 | PQ152720 | | PQ202723 | | PQ152744 | Sarawi et al. 2025a | | |
| 80 | *Echinoderma asperum* | | KaiR170 | | Germany | | PQ152686 | PQ152718 | | PQ202721 | | PQ152745 | Sarawi et al. 2025a | | |
| **Part B: Additional taxa in the ITS-only dataset (N=96)** | | | | | | | | | | | | | |  | |
| **No.** | | **Taxon** | | **Voucher** | | **Location** | | | **ITS** | | **References** | | | |  |
| 1 | | *L. albofloccosa* | | MAA-01 (HT) | | India | | | OP954870 | | Ahamed et al. 2023 | | | |  |
| 2 | | *L. alopochroa* | | MFLU 090178 | | Thailand, Chiang Mai | | | HQ647294 | | Sysouphanthong et al. 2011 | | | |  |
| 3 | | *L. amplicystidiata* | | HMAS53632 (PT) | | China: Tibet | | | JN203141 | | Liang 2012 | | | |  |
| 4 | | *L. andegavensis* | | Roux 2121 | | France, Coudre | | | AY176461 | | Vellinga 2003 | | | |  |
| 5 | | *L. aspericeps* | | HKAS46051 | | USA, Arkansas | | | MK651620 | | Li X et al. 2025 | | | |  |
| 6 | | *L. asperula* | | iNaturalist # 8607856 | | USA: Indiana | | | MN906140 | | Unpublished GenBank | | | |  |
| 7 | | *L. aurantiopilea* | | LAH37661 (HT) | | Pakistan | | | NR_198382 | | Asif et al. 2024 | | | |  |
| 8 | | *L. aureofulvella* | | MFLU 090183 (HT) | | Thailand: Chiang Mai | | | HQ647293 | | Sysouphanthong et al. 2011 | | | |  |
| 9 | | *L. babruka* | | K(M)155991 (HT) | | India | | | PQ152691 | | Sarawi et al. 2025a | | | |  |
| 10 | | *L. badiopurpurea* | | EMB15952005 (HT) | | Italy | | | PQ152693 | | Sarawi et al. 2025a | | | |  |
| 11 | | *L. bahawalnagarensis* | | LAH37787 (HT) | | Pakistan | | | NR_198421 | | Asif et al. 2024 | | | |  |
| 12 | | *L. bengalensis* | | Iqbal 825 (HT) | | Bangladesh: Dhaka | | | KU563148 | | Hosen et al. 2016 | | | |  |
| 13 | | *L. boertmannii* | | C-F-13679 (HT) | | Denmark | | | PQ152713 | | Sarawi et al. 2025a | | | |  |
| 14 | | *L. brunneoaurantia* | | UEH-F0006 (HT) | | Pakistan | | | OR464184 | | Azeem et al. 2024 | | | |  |
| 15 | | *L. brunneodisca* | | GUBH 20381 (HT) | | India | | | ON935795 | | Niranjan et al. 2023 | | | |  |
| 16 | | *L. brunneogranulosa* | | LAH:36803 (HT) | | Pakistan | | | NR_184481 | | Asif et al. 2022 | | | |  |
| 17 | | *L. brunneopileata* | | LAH37842 (HT) | | Pakistan | | | NR_198496 | | Rehman et al. 2024 | | | |  |

Supplementary Table 1. (Continued)

| **No.** | **Taxon** | **Voucher** | **Location** | **ITS** | **References** |
| --- | --- | --- | --- | --- | --- |
| 18 | *L. carinii* | EMB13282005 (Epitype) | Italy | PQ152699 | Sarawi et al. 2025a |
| 19 | *L. castaneidisca* | ecv 2411 (UC) | USA, California | AF391063 | Vellinga 2001b |
| 20 | *L. chiangraiensis* | MFLU 20–0197 (HT) | Thailand | MT020094 | Hyde et al. 2020 |
| 21 | *L. cholistanensis* | LAH 35831 (HT) | Pakistan | NR_173802 | Bashir et al. 2020 |
| 22 | *L. cingulum* | 20-IX-1995, M. Enderle | Germany, Bavaria | AY176359 | Vellinga 2004a |
| 23 | *L. citrophylla* | HNL502963 | Laos, Oudomxay | KX711969 | Sysouphanthong et al. 2020a |
| 24 | *L. clypeolarioides* | MCVE:16904 |  | FJ998407 | Unpublished GenBank |
| 25 | *L. coloratipes* | SAV F-3213 (HT) | Spain: Morella | KC900377 | Vizzini et al. 2013 |
| 26 | *L. condylospora* | MFLU 090048 (HT) | Thailand: Chiang Mai | JN224822 | Sysouphanthong et al. 2020b |
| 27 | *L. cremea* | OKA-TR11019 (HT) | Turkey: Denizli | OL630458 | Kaygusuz 2022 |
| 28 | *L. cristatoides* | 5-IX-1996, Huijser s.n. | Netherlands | AY176363 | Vellinga 2004a |
| 29 | *L. cylindrocystidia* | MFLU 12-2035 (HT) | Thailand: Chiang Mai | MW251841 | Tibpromma et al. 2017 |
| 30 | *L. cystophoroides* | ecv 2142 (L) | France, | AF391031 | Vellinga 2001a |
| 31 | *L. echinella* | 4-X-1998, Huijser s.n. | Belgium, Luxembourg e | AY176366 | Vellinga 2004a |
| 32 | *L. efibulis* | K(M)188857 (HT) | United Kingdom | PQ152714 | Sarawi et al. 2025a |
| 33 | *L. elseae* | AH40487 (HT) | Spain | NR_158471 | Caballero et al. 2015 |
| 34 | *L. eurysperma* | MFU0900035 (HT) | Thailand, Chiang Mai | HQ718462 | Sysouphanthong et al. 2012 |
| 35 | *L. exocarpi* | PERTH:08944946 | Australia | MT571655 | Unpublished GenBank |
| 36 | *L. faiae-bravae* | BCN-IC11111501 (HT) | Portugal: Guarda | PP622390 | Paz and Lavoise 2024 |
| 37 | *L. flavocarpa* | MFLU 10-0581 (HT) | Thailand, Chiang Mai | MW251842 | Tibpromma et al. 2017 |
| 38 | *L. flavostipitata* | SeSa182 (PT) | Benin | PP594568 | Sarawi et al. 2025b |
| 39 | *L. forquignonii* | ecv 2284 (L) | Netherlands | AY176370 | Vellinga 2004a |
| 40 | *L. geogenia* | MEL:2358502 (HT) | Australia | NR_120304 | Lebel and Vellinga 2013 |
| 41 | *L. haroonabadensis* | LAH36801 (HT) | Pakistan | NR_173325 | Niazi et al. 2021 |
| 42 | *L. helveola* | PUL:00030876 | USA: Indiana | OM522689 | Unpublished GenBank |
| 43 | *L. himalayensis* | LAH 230810 (HT) | Pakistan | NR_155308 | Razaq et al. 2012 |

Supplementary Table 1. (Continued)

| **No.** | **Taxon** | **Voucher** | **Location** | **ITS** | **References** |
| --- | --- | --- | --- | --- | --- |
| 44 | *L. hymenoderma* | ecv 2017 (L) | Netherlands | AF391028 | Vellinga 2001a |
| 45 | *L. iberica* | JMV800332 (HT) | Spain | KT315646 | Vidal et al. 2015 |
| 46 | *L. ignicolor* | 17-X-1999, Huijser | Netherlands | AY176472 | Vellinga 2003 |
| 47 | *L. inconspicua* | SAPA:1097 |  | LC683197 | Unpublished GenBank |
| 48 | *L. kammala* | TLE1079 | Australia | OL653134 | Unpublished GenBank |
| 49 | *L. keralensis* | ZGCJN19 (HT) | India | OR610708 | Pious et al. 2025 |
| 50 | *L. lahorensis* | T18 (HT) | Pakistan | KT182475 | Qasim et al. 2016 |
| 51 | *L. lepida* | MCVE:727 |  | FJ998392 | NCBI, unpublished |
| 52 | *L. luteophylla* | H.V. Smith 284 | USA, Michigan | AY176475 | Vellinga 2003 |
| 53 | *L. maculans* | JMB080509_18 | USA, Tennessee | HM222939 | Birkebak et al. 2011 |
| 54 | *L. maerimensis* | MFLU 12-2036 (HT) | Thailand | MW251839 | Tibpromma et al. 2017 |
| 55 | *L. mandarina* | HKAS 50028 (HT) | China, Yunnan | KM214811 | Liang 2016 |
| 56 | *L. nigrescentipes* | G. Riousset 93051001 | France | AY176382 | Vellinga 2003 |
| 57 | *L. nigrosquamosa* | HKAS 33874 (HT) | China, Sichuan | JN203140 | Liang and Yang 2012 |
| 58 | *L. ochraceodisca* | PAM02111802 | France | KT315648 | Vidal et al. 2015 |
| 59 | *L. omninoflava* | HKAS 106734 (HT) | China, Yunnan | MN810157 | Hou and Ge 2020 |
| 60 | *L. pakistanensis* | LAH37846 (HT) | Pakistan | OQ954776 | Rehman et al. 2024 |
| 61 | *L. pallidiochracea* | HKAS 45579 (HT) | China: Tibet | NR_158462 | Liang and Yang 2011 |
| 62 | *L. parvannulata* | MCVE:2297 |  | FJ998396 | Unpublished GenBank |
| 63 | *L. phaeoderma* | ecv 3000 (UC) | USA, California | GQ203810 | Vellinga 2010 |
| 64 | *L. pleurocystidiata* | MFLU 20–0196 (HT) | Thailand, Chiang Rai | MT020093 | Hyde et al. 2020 |
| 65 | *L. poliochloodes* | ecv3877 | Thailand, Chiang Mai | HQ647296 | Sysouphanthong et al. 2011 |
| 66 | *L. pongduadensis* | MFU090184 (PT) | Thailand: Chiang Mai | HQ718461 | Sysouphanthong et al. 2012 |
| 67 | *L. psalion* | WU 5152 (HT) | Austria | MG581687 | Vizzini et al. 2019 |
| 68 | *L. pseudoasperula* | C-F-13685 (HT) | Denmark | PQ152710 | Sarawi et al. 2025a |
| 69 | *L. pseudofelina* | MCVE:3553 |  | FJ998398 | Unpublished GenBank |

Supplementary Table 1. (Continued)

| **No.** | **Taxon** | **Voucher** | **Location** | **ITS** | **References** |
| --- | --- | --- | --- | --- | --- |
| 70 | *L. pyrochroa* | ecv 2006 (L) | Netherlands | AY176477 | Vellinga 2003 |
| 71 | *L. recondita* | TR gmb 01482 (HT) | Netherlands | NR_173167 | Vizzini et al. 2019 |
| 72 | *L. rhodophylla* | ecv 3026 (HT) | USA: California | NR_119624 | Vellinga 2006 |
| 73 | *L. rufobrunnea* | MAA23-06 (HT) | India | PP331855 | Ahamed et al. 2024 |
| 74 | *L. sanguineofracta* | TO-HG2916 (HT) | Italy | NR_166796 | Vizzini et al. 2020 |
| 75 | *L. sayanensis* | LE 312933 (HT) | Russia | NR_182944 | Crous et al. 2021 |
| 76 | *L. scaberula* | ecv 2595 (HT) | USA, California, | NR_119449 | Vellinga 2001a |
| 77 | *L. sindhudeltana* | LAH37025 (HT) | Pakistan | OM987446 | Haqnawaz et al. 2022 |
| 78 | *L. smurfiorum* | JMV-8000331 (HT) | Spain | KT315647 | Vidal et al. 2015 |
| 79 | *L. sosuensis* | CA3 (HT) | Dominican, Sosua | NR_184875 | Justo et al. 2015 |
| 80 | *L. sp.* | iNaturalist # 206190502 | USA, California | PV587857 | Unpublished GenBank |
| 81 | *L. sp.* | ecv 2609 | USA, California | AY176483 | Vellinga 2003 |
| 82 | *L. speciosa* | MCVE:9565 |  | FJ998402 | Unpublished GenBank |
| 83 | *L. subalba* | ecv 2242 | Netherlands | AY176489 | Vellinga 2003 |
| 84 | *L. subcastanea* | HKAS 45633 (HT) | China, Tibet | KM214812 | Liang 2016 |
| 85 | *L. subgranulosa* | ANGE253 | Dominican, Sosua | KR022007 | Justo et al. 2015 |
| 86 | *L. sublaevigata* | PAM02081205 | France | KT315650 | Vidal et al. 2015 |
| 87 | *L. subvenenata* | 170927-68 (HT) | China, Yunnan | MK411234 | Zhang et al. 2019 |
| 88 | *L. thailandica* | MFLU 090120 (HT) | Thailand, Chiang Mai | JN224824 | Sysouphanthong et al. 2012 |
| 89 | *L. thiersii* | ecv 2589 | USA, California | GQ203817 | Vellinga 2010 |
| 90 | *L. umbrosa* | S.D. Russell HRL2084 | Canada | MH979461 | Unpublished GenBank |
| 91 | *L. vellingana* | MCR09 (HT) | Pakistan | HE974764 | Rizwana et al. 2012 |
| 92 | *L. venenata* | HKAS101874 (HT) | China, Hubei | NR_160618 | Cai et al. 2018 |
| 93 | *L. viridigleba* | Trappe 9493 (HT) | Laos | JX014271 | Ge and Smith 2013 |
| 94 | *L. xanthophylla* | ecv 2240 (L) | Netherlands | AY176405 | Vellinga 2004a |
| 95 | *L. squamulodiffracta* | CA21 (HT) | Dominican, Sosua | NR_184876 | Justo et al. 2015 |

Supplementary Table 1. (Continued)

| 96 | *Echinoderma perplexum* | KaiR1713 (Epitype) | Germany | PP594623 | Sarawi et al. 2025b |
| --- | --- | --- | --- | --- | --- |

**Additional reference (not included in the reference of text)**

Ahamed M, Verma K, Dutta AK, Sharma YP (2023) *Lepiota albofloccosa*, a new species in sect. *Lepiota* (Agaricaceae, Agaricales) from Northwestern Himalayas of Jammu and Kashmir, India. Phytotaxa 607(1): 72–84. https://doi.org/10.11646/phytotaxa.607.1.6

Ahamed M, Verma K, Dutta AK, Sharma YP (2024) A novel species of *Lepiota* sect. *Lepiota* (Agaricaceae) from Jammu and Kashmir, India. Taiwania 69(4): 522–529. https://doi.org/10.6165/tai.2024.69.522

Asif M, Izhar A, Haqnawaz M, Niazi AR, Khalid AN (2022) *Lepiota* *brunneogranulosa* (Agaricaceae): a new species from Punjab, Pakistan, based on integrated taxonomy. Acta Botanica Brasilica 36: e2021abb0382. https://doi.org/10.1590/0102-33062021abb0382

Asif M, Izhar A, Niazi AR, Khalid AN, Saba M (2024) Mycological surveys reveal two new species of genus *Lepiota* (Agaricaceae) from the semi-arid climatic region of Punjab, Pakistan. Mycological Progress 23: 20. https://doi.org/10.1007/s11557-024-01958-0

Azeem M, Kiran M, Jabeen S (2024) Molecular phylogeny and morphological characterization revealed *Lepiota* *brunneoaurantia*, a new species in *L.* sect. *Stenosporae* from Margalla Hills, Pakistan. Phytotaxa 636(1): 61–73. https://doi.org/10.11646/phytotaxa.636.1.5

Bashir H, Usman M, Khalid AN (2020) *Lepiota* *cholistanensis* a new species of *Lepiota* (Agaricaceae: Basidiomycota) from Cholistan desert, Pakistan. Phytotaxa 455(4):267–276. https://doi.org/10.11646/phytotaxa.455.4.4

Birkebak JM, Vellinga EC, Franco-Molano AE, Wood MG, Matheny PB (2011) *Lepiota* *maculans*, an Unusual Mushroom Rediscovered after 105 years. Southeastern Naturalist (Steuben, ME) 10(2): 267–274. https://doi.org/10.1656/058.010.0207

Caballero A, Vizzini A, Munoz G, Contu M, Ercole E (2015) *Lepiota* *elseae* (Agaricales, Agaricaceae), a new species of section *Lepiota* from Spain. Phytotaxa 201(3): 188–196. https://doi.org/10.11646/phytotaxa.201.3.2

Cai Q, Chen ZH, He ZM, Luo H, Yang ZL (2018) *Lepiota* *venenata*, a new species related to toxic mushroom in China. Journal of Fungal Research 16(2): 63–69.

Crous PW, Osieck ER, Jurjević Ž, et al. (2021) Fungal Planet description sheets: 1284-1382. Persoonia 47: 178–374. https://doi.org/10.3767/persoonia.2021.47.06.

Haqnawaz M, Niazi AR, Usman M, Khalid AN (2022) *Lepiota* *sindhudeltana* sp. nov. (Agaricaceae; Basidiomycota) from Punjab, Pakistan. Phytotaxa 550(3): 253–262. https://doi.org/10.11646/phytotaxa.550.3.5

Hosen MI, Li TH, Ge ZW, Vellinga EC (2016) *Lepiota* *bengalensis*, a new species of *Lepiota* section *Lilaceae* from Bangladesh. Sydowia 68: 187–192. https://doi.org/10.12905/0380.sydowia68-2016-0187

Hyde KD, Jeewon R, Chen YJ, Bhunjun CS, Calabon MS, Jiang HB, Lin CG, Norphanphoun C, Sysouphanthong P, Pem D, et al. 2020. The numbers of fungi: is the descriptive curve flattening? Fungal Diversity 103: 219–271. https://doi.org/10.1007/s13225-020-00458-2

Kaygusuz O (2022) *Lepiota* *cremea*, a novel species of *Lepiota* sect. *Lepiota* (Agaricaceae s.l., Agaricales) from Turkey. Phytotaxa 555(4): 291–300. https://doi.org/10.11646/phytotaxa.555.4.2

Kropp BR, Albee-Scott S, Castellano MA, Trappe JM (2012) *Cryptolepiota*, a new sequestrate genus in the Agaricaceae with evidence for adaptive radiation in western North America. Mycologia 104(1): 164–74. https://doi.org/10.3852/11-046.

Liang JF (2016) Taxonomy and phylogeny in *Lepiota* sect. *Stenosporae* from China. Mycologia 108(1): 56–69. https://doi.org/10.3852/15-105

Liang JF, Yang ZL, Xu JP, Ge ZW (2010) Two new unusual *Leucoagaricus* species (Agaricaceae) from tropical China with blue-green staining reactions. Mycologia 102(5): 1141–1152.

Niazi AR, Asif M, Izhar A, Khalid AN (2021) A new species of *Lepiota* (Agaricaceae) from Punjab, Pakistan. Phytotaxa 511(2): 163–174. https://doi.org/10.11646/phytotaxa.511.2.4

Niranjan R, Talukdar M, Bagchi T, Dutta AK (2023) *Lepiota* *brunneodisca*, a new taxon of *Lepiota* Sect. *Ovisporae* from India. Biology Bulletin of the Russian Academy of Sciences 50: 1211–1217. https://doi.org/10.1134/S1062359023602720

Pious JR, Kumar TKA (2025) *Lepiota* *keralensis*, a new species with epithelial pileal covering. Nordic Journal of Botany 5: e04648. https://doi.org/10.1111/njb.04648

Qasim T, Khalid AN, Vellinga EC (2016) A new species of *Lepiota*, *Lepiota* *lahorensis*, from Lahore, Pakistan. Turk J Bot. 40(4): 419–426. https://doi.org/10.3906/bot-1507-31

Razaq A, Khalid AN, Vellinga EC (2012) *Lepiota himalayensi*s (Basidiomycota, Agaricales), a new species from Pakistan. Mycotaxon 121: 319–325. http://dx.doi.org/10.5248/121.319

Rehman A, Usman M, Afshan NUS, Khalid AN (2024) Taxonomy and phylogeny reveal two novel species of genus *Lepiota* (Agaricaceae, Agaricales) from Punjab, Pakistan. Plant Systematics and Evolution 310: 8. [1–12] https://doi.org/10.1007/s00606-02401891-5

Rizwana N, Khalid AN, Hanif M, Razaq A (2012) *Lepiota* *vellingana* sp. nov. (Basidiomycota, Agaricales) a new species from Lahore, Pakistan. Mycol Prog. 12:727–732.

Stallman JK, Hemmes DE, Hynson NA, Shintaku MH (2020) *Lepiota* *punaensis* sp. nov. from Hawai'i Island, and a discussion of *L. elaiophylla*. Mycotaxon 135(3): 471–489. https://doi.org/10.5248/135.471

Sysouphanthong P, Hyde KD, Chukeatirote E, Bahkali AH, Vellinga EC (2011) *Lepiota* (Agaricales) in northern Thailand – 1. *L.* section *Stenosporae*. Mycotaxon 117: 53–85. http://dx.doi.org/10.5248/117.53

Sysouphanthong P, Hyde KD, Chukeatirote E, Bahkali AH, Vellinga EC (2012) *Lepiota* (Agaricales) in northern Thailand-2 Lepiota section *Lepiota*. Cryptogamie Mycologie 33(1): 25–42. https://doi.org/10.7872/crym.v33.iss1.2012.025

Sysouphanthong P, Thongklang N, Suwannapoom C, Nuangmek W, Hyde KD (2020a) *Lepiota* Section *Stenosporae* (Agaricaceae): Two New Records to Lao People's Democratic Republic. Chiang Mai Journal of Science 47(1): 49–56.

Sysouphanthong P, Thongklang N, Karunarathna SC, Mortimer PE, Hyde KD, Vellinga EC (2020b) *Lepiota condylospora*, a new species with nodulose spores in section *Lilaceae* from northern Thailand. Phytotaxa 455(2): 61–69. https://doi.org/10.11646/phytotaxa.455.2.1

Tibpromma S, Hyde KD, Jeewon R, et al. (2017) Fungal diversity notes 491–602: taxonomic and phylogenetic contributions to fungal taxa. Fungal Diversity 83: 1–261. https://doi.org/10.1007/s13225-017-0378-0

Vellinga EC (2001a) Studies in *Lepiota* III - Some species from California, USA. Mycotaxon 80: 285–295.

Vellinga EC (2001b) Studies in *Lepiota* IV - *Lepiota* *cristata* and *Lepiota* *castaneidisca*. Mycotaxon 80: 297–306.

Vellinga EC (2006) Lepiotaceous fungi in California, U.S.A. – 2. *Lepiota* *rhodophylla* sp. nov. Mycotaxon 98: 205–211.

Vellinga EC (2010) *Lepiota* in California: species with a hymeniform pileus covering. Mycologia 102(3): 664–675. https://doi.org/10.3852/09-180

Vellinga EC, Sysouphanthong P, Hyde KD (2011) The family Agaricaceae: phylogenies and two new white-spored genera. Mycologia 103(3): 494–509. https://doi.org/10.3852/10-204

Vizzini A, Ercole E, Voyron S (2020) *Lepiota* *sanguineofracta* (Basidiomycota, Agaricales), a new species with a hymeniform pileus covering from Italy. Mycological Progress 13: 683–690. https://doi.org/10.1007/s11557-013-0950-2

Vizzini A, Liang JF, Jančovičová S, Adamčík S, Ercole E, Contu M, Yang ZL, Vellinga EC (2014) *Lepiota* *coloratipes*, a new species for *Lepiota* *rufipes* ss. Auct. europ. non ss. orig. Mycological Progress 13: 171–179. https://doi.org/10.1007/s11557-013-0905-7

Vizzini A, Tatti A, Huijser HA, Liang JF, Ercole E (2019) Looking for *Lepiota* *psalion* Huijser & Vellinga (Agaricales, Agaricaceae). MycoKeys 52: 45–69. https://doi.org/10.3897/mycokeys.52.34021
