# Supplementary material for: Studies in Lepiota (Agaricales, Verrucosporaceae): supporting the merger of Chamaemyces into Lepiota and proposing two new species
Source: MycoKeys. 2026 May 28;133:103–25. doi: 10.3897/mycokeys.133.186351 (PMC13237568; doi:10.3897/mycokeys.133.186351)
Supplement: Supplementary material 5 — Phylogenetic tree [file mycokeys-133-103-s005.doc]

**Supplementary File 5. Phylogenetic tree based on the combined dataset of ITS, LSU, *rpb2*, and *tef1*‑α sequences, and the list of the vouchers, location and GenBank accession numbers.
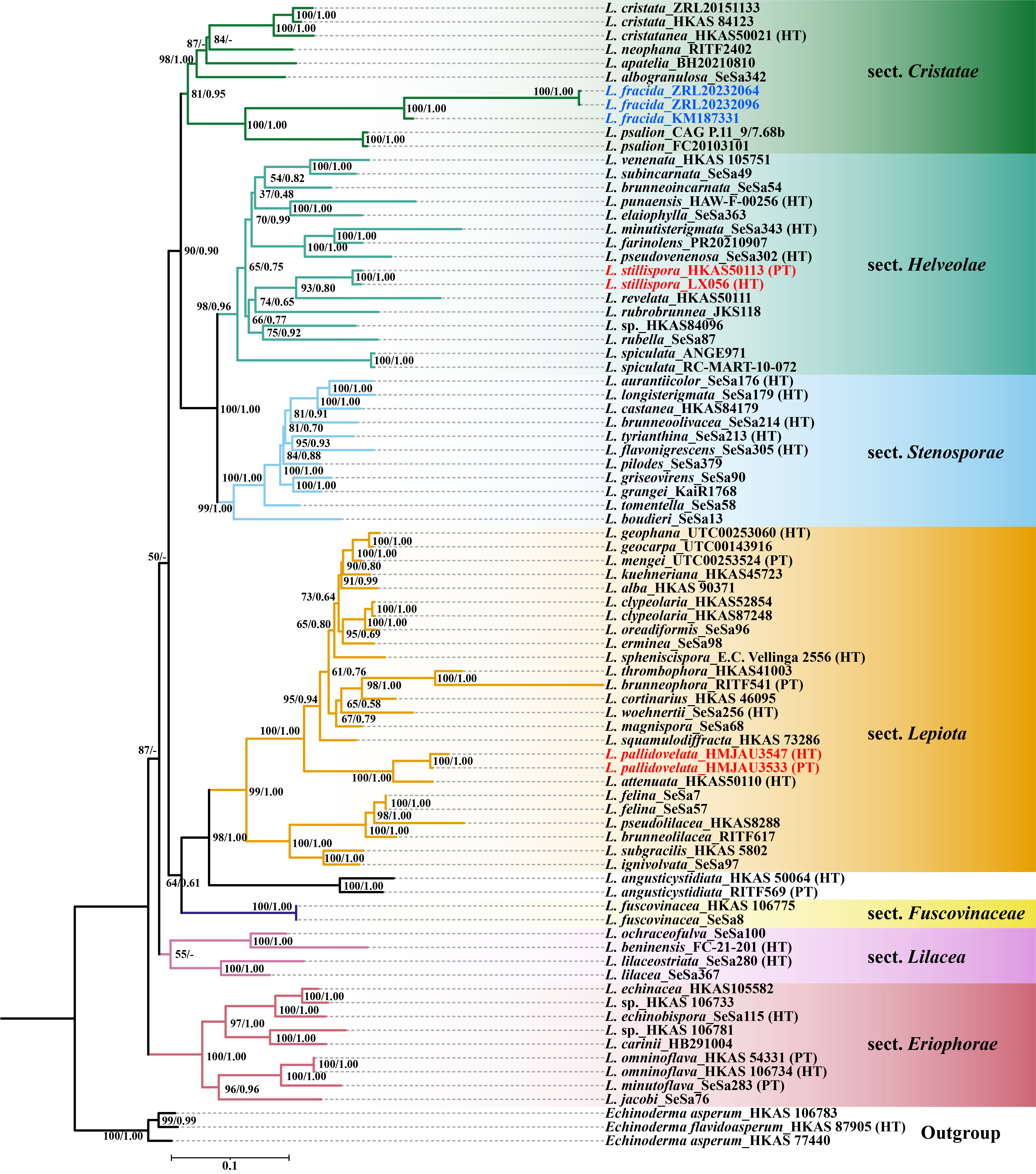
**

| **No.** | **Taxon** | **Voucher** | **Location** | **ITS** | **LSU** | ***rpb2*** | ***tef1*‑α** |
| --- | --- | --- | --- | --- | --- | --- | --- |
| 1 | *L. alba* | HKAS 90371 | China, Xinjiang | MN810115 | MN810075 | MN820946 | MN820930 |
| 2 | *L. attenuata* | HKAS50110 (HT) | China, Yunnan | EU681776 | GU199354 | MK705816 |  |
| 3 | *L. clypeolaria* | HKAS52854 | USA, Arkansas | MK651622 | MK685363 | MK705819 |  |
| 4 | *L. clypeolaria* | HKAS87248 | China, Yunnan | MN810123 | MN810080 | MN820941 | MN820932 |
| 5 | *L. cortinarius* | HKAS 46095 | China, Tibet | EU416306 | EU416307 | MK705820 |  |
| 6 | *L. erminea* | SeSa98 | Germany | PP594552 | PP594670 | PP841276 |  |
| 7 | *L. geocarpa* | UTC00143916 | USA, Utah | HQ020412 | EU130550 | MN820945 | MN820935 |
| 8 | *L. geophana* | UTC00253060 (HT) | USA | HQ020411 | HQ020421 | MN820944 | MN820934 |
| 9 | *L. kuehneriana* | HKAS45723 | China, Tibet | GU199360 | GU199358 | MK705821 |  |
| 10 | *L. magnispora* | SeSa68 | Austria | PP594541 | PP594657 | PP841263 | MN820937 |
| 11 | *L. mengei* | UTC00253524 (PT) | USA, Utah | MN810131 | MN810082 | MN820942 | MN820936 |
| 12 | *L. oreadiformis* | SeSa96 | Germany | PP594550 | PP594668 | PP841274 |  |
| 13 | *L. squamulodiffracta* | HKAS 73286 | China, Yunnan | MN810120 | MN810078 | MN820947 | MN820938 |
| 14 | *L. spheniscispora* | ecv 2556 (HT) | USA, California | NR_119448 | AY176404 | HM488813 | HM488951 |
| 15 | *L. thrombophora* | HKAS41003 | China, Hainan | EU681780 | MK651687 | MK705829 |  |
| 16 | *L. brunneophora* | RITF541 (PT) | China | MK651649 | MK685364 | MK705830 |  |
| 17 | *L. woehnertii* | SeSa256 (HT) | Benin | PP594583 | PP594704 | PP841212 |  |
| 18 | *L. pallidovelata* | HMJAU3547 (HT) | China | MK651643 | MK651684 | PX913186 |  |
| 19 | *L. pallidovelata* | HMJAU3533 (PT) | China | MK651644 | MK651685 | PX913187 |  |
| 20 | *L. ignivolvata* | SeSa97 | Germany | PP594551 | PP594669 | PP841275 |  |
| 21 | *L. subgracilis* | HKAS 5802 | China, Jilin | EU416290 | EU416291 | MK705815 |  |
| 22 | *L. felina* | SeSa57 | Germany | PP594535 | PP594651 | PP841258 |  |
| 23 | *L. felina* | SeSa7 | Germany | PP594523 | PP594633 | PP841264 |  |
| 24 | *L. pseudolilacea* | HKAS8288 | China, Jilin | EU416304 | EU416305 | MK705808 |  |
| 25 | *L. brunneolilacea* | RITF617 | USA, Arkansas | MK651609 | MK651664 | MK705806 |  |
| 26 | *L. angusticystidiata* | HKAS 50064 (HT) | China, Yunnan | KP177192 | KP177198 | MK705838 |  |
| 27 | *L. angusticystidiata* | RITF569 (PT) | China, Yunnan | KP177194 | KP177199 | MK705839 |  |

Table (Continued)

| **No.** | **Taxon** | **Voucher** | **Location** | **ITS** | **LSU** | ***rpb2*** | ***tef1*‑α** |
| --- | --- | --- | --- | --- | --- | --- | --- |
| 28 | *L. lilacea* | SeSa367 | Germany | PP594612 | PP594735 | PP841244 |  |
| 29 | *L. ochraceofulva* | SeSa100 | Germany | PP594554 | PP594672 | PP841178 |  |
| 30 | *L. beninensis* | FC-21-201 (HT) | Benin | PP594630 | PP594755 | PP841161 |  |
| 31 | *L. lilaceostriata* | SeSa280 (HT) | Benin | PP594588 | PP594709 | PP841217 |  |
| 32 | *L. fuscovinacea* | HKAS 106775 | Germany | MN810125 |  | MN820949 | MN820912 |
| 33 | *L. fuscovinacea* | SeSa8 | Germany | PP594524 | PP594634 | PP841268 |  |
| 34 | *L. neophana* | RITF2402 | China | MK651599 | MK651661 | MK705837 | GQ375551 |
| 35 | *L. psalion* | CAG P.11_9/7.68b | Italy | MG581689 | MG581700 |  | MG597230 |
| 36 | *L. psalion* | FC20103101 | Germany | PP594616 | PP594743 | PP841160 | MG597229 |
| 37 | *L. albogranulosa* | SeSa342 | Benin | PP594605 | PP594728 | PP841237 |  |
| 38 | *L. cristatanea* | HKAS50021 (HT) | China, Yunnan | EU081952 | MK651659 | MK705836 |  |
| 39 | *L. apatelia* | BH20210810 | Germany | PP594619 | PP594746 | PP841157 |  |
| 40 | *L. cristata* | ZRL20151133 | China | LT716026 | KY418841 | KY418992 | KY419048 |
| 41 | *L. cristata* | HKAS 84123 | China, Sichuan | MN810124 | MN810081 | MN820948 | MN820911 |
| 42 | *L. fracida* | KM187331 | United Kingdom | PP763181 | PP763097 | PP829104 | PP808559 |
| 43 | *L. fracida* | ZRL20232064 |  | PV607755 | PV607719 | PV614752 | PV614790 |
| 44 | *L. fracida* | ZRL20232096 |  | PV607756 | PV607720 | PV614753 | PV614791 |
| 45 | *L. brunneoincarnata* | SeSa54 | Germany | OL527694 | PP594648 | PP841256 |  |
| 46 | *L. elaiophylla* | SeSa363 | Germany | PP594611 | PP594734 | PP841243 |  |
| 47 | *L. farinolens* | PR20210907 | Germany | PP594618 | PP594745 | PP841167 |  |
| 48 | *L. spiculata* | RC-MART-10-072 | France | PP594628 | PP594753 | PP841168 |  |
| 49 | *L. spiculata* | ANGE971 |  | MK696156 | MK696155 | MK696576 | MK696577 |
| 50 | *L. punaensis* | HAW-F-00256 (HT) | USA, Hawaii | NR_173815 | NG_153999 | OM215203 |  |
| 51 | *L. revelata* | HKAS50111 | USA, Arkansas | MK651616 | MK651669 | MK705813 |  |
| 52 | *L. rubella* | SeSa87 | Germany | PP594544 | PP594661 | PP841269 |  |
| 53 | *L. rubrobrunnea* | JKS118 | USA, Hawaii | MK412583 | OM203354 | OM215204 |  |

Table (Continued)

| **No.** | **Taxon** | **Voucher** | **Location** | **ITS** | **LSU** | ***rpb2*** | ***tef1*‑α** |
| --- | --- | --- | --- | --- | --- | --- | --- |
| 54 | *L. stillispora* | LX056 (HT) | China, Guangdong | PX423621 | PX423622 | PX913184 |  |
| 55 | *L. stillispora* | HKAS50113 (PT) | China, Yunnan | PX105037 | PX105041 | PX913185 |  |
| 56 | *L. subincarnata* | SeSa49 | Germany | PP594533 | PP594646 | PP841254 |  |
| 57 | *L. venenata* | HKAS 105751 | China, Guangxi | MN810128 |  | MN820958 | MN820927 |
| 58 | *L. minutisterigmata* | SeSa343 (HT) |  | PP594606 | PP594729 | PP841238 |  |
| 59 | *L. pseudovenenosa* | SeSa302 (HT) |  | PP594593 | PP594714 | PP841223 |  |
| 60 | *L. castanea* | HKAS84179 | China, Sichuan | MN810119 | MN810077 | MN820960 | MN820914 |
| 61 | *L. grangei* | KaiR1768 | Germany | PP594624 | PP594638 | PP841166 |  |
| 62 | *L. griseovirens* | SeSa90 | Germany | PP594547 | PP594664 | PP841271 |  |
| 63 | *L. pilodes* | SeSa379 | Germany | PP594614 | PP594736 | PP841247 |  |
| 64 | *L. tomentella* | SeSa58 | Germany | PP594536 | PP594652 | PP841259 |  |
| 65 | *L. aurantiicolor* | SeSa176 (HT) | Benin | PP594563 | PP594681 | PP841188 |  |
| 66 | *L. brunneoolivacea* | SeSa214 (HT) | Benin | PP594574 | PP594693 | PP841201 |  |
| 67 | *L. flavonigrescens* | SeSa305 (HT) | Benin | PP594596 | PP594717 | PP841226 |  |
| 68 | *L. longisterigmata* | SeSa179 (HT) | Benin | PP594565 | PP594683 | PP841190 |  |
| 69 | *L. tyrianthina* | SeSa213 (HT) | Benin | PP594573 | PP594692 | PP841200 |  |
| 70 | *L. boudieri* | SeSa13 | Austria | OL527685 | PP594636 | PP841186 |  |
| 71 | *L. carinii* | HB291004 | Germany | PQ152700 | PQ152728 | PQ202731 |  |
| 72 | *L. echinacea* | HKAS105582 | China, Gansu | MN810155 | MN810104 | MN820954 | MN820928 |
| 73 | *L. jacobi* | SeSa76 | Germany | PP594543 | PP594659 | PP841266 |  |
| 74 | *L. omninoflava* | HKAS 54331 (PT) | China, Yunnan | MN810141 | MN810093 | MN820952 | MN820924 |
| 75 | *L. omninoflava* | HKAS 106734 (HT) | China, Yunnan | MN810157 | MN810092 | MN820951 | MN820923 |
| 76 | *L. echinobispora* | SeSa115 (HT) | Benin | PP594560 | PP594678 | PP841184 |  |
| 77 | *L. minutoflava* | SeSa283 (PT) | Benin | PP594590 | PP594711 | PP841219 |  |
| 78 | *L.* sp. | HKAS 84096 | China, Yunnan | MN810116 | MN810076 | MN820956 | MN820922 |
| 79 | *L.* sp. | HKAS 106733 | China, Yunnan | MN810156 | MN810105 | MN820955 | MN820929 |

Table (Continued)

| **No.** | **Taxon** | **Voucher** | **Location** | **ITS** | **LSU** | ***rpb2*** | ***tef1*‑α** |
| --- | --- | --- | --- | --- | --- | --- | --- |
| 80 | *L.* sp. | HKAS 106781 | China, Yunnan | MN810132 | MN810086 | MN820950 | MN820939 |
| 81 | *Echinoderma asperum* | HKAS 77440 | China, Yunnan | MN810145 | MN810096 | MN820963 | MN820906 |
| 82 | *Echinoderma asperum* | HKAS 106783 | North Macedonia | MN810133 | MN810088 | MN820967 | MN820902 |
| 83 | *Echinoderma flavidoasperum* | HKAS 87905 (HT) | China, Yunnan | NR_171246 | NG_073786 | MN820969 | MN820903 |
