# Supplementary figures and images for: Studies in Lepiota (Agaricales, Verrucosporaceae): supporting the merger of Chamaemyces into Lepiota and proposing two new species
Source: MycoKeys. 2026 May 28;133:103–25. doi: 10.3897/mycokeys.133.186351 (PMC13237568; doi:10.3897/mycokeys.133.186351)

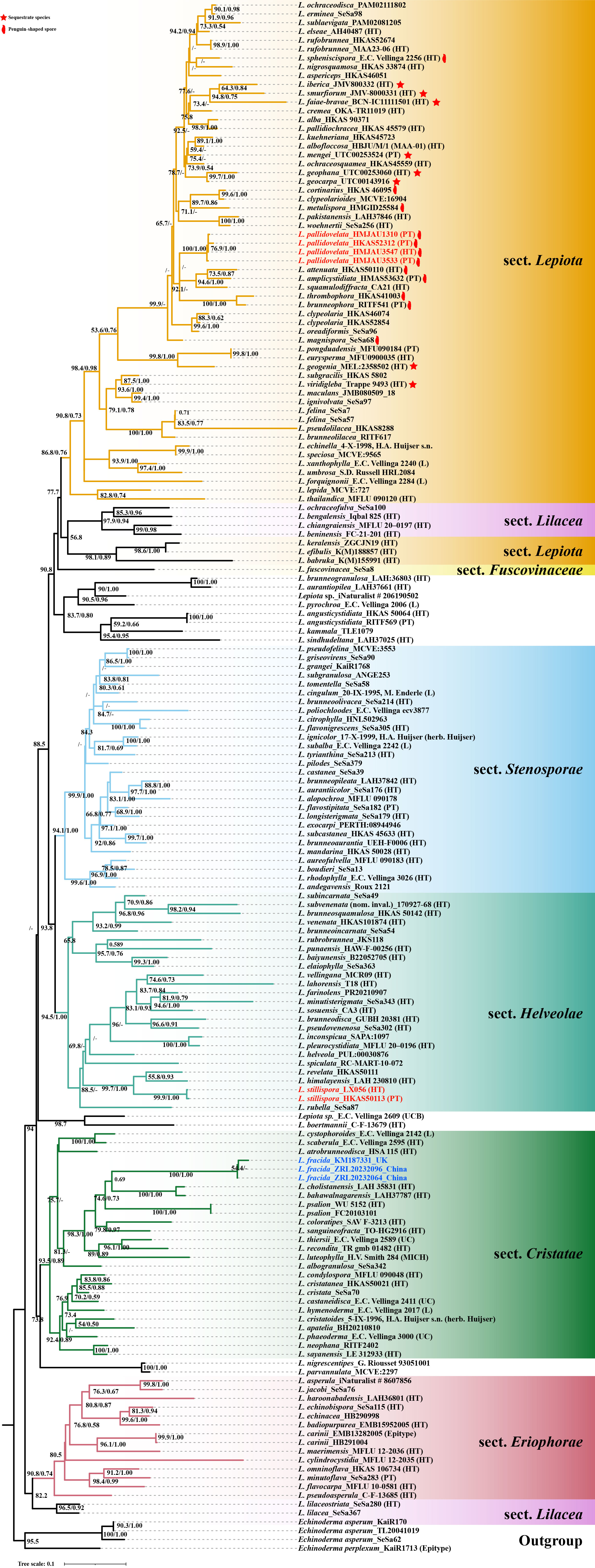

Supplement: Supplementary material 6 — Phylogenetic tree [file mycokeys-133-103-s006.jpg]
